# Nanoparticle Strategies for Bone Metastasis Immunotherapy: Targeting, Immune Reprogramming and Combination Therapy

**DOI:** 10.3390/pharmaceutics18050571

**Published:** 2026-05-04

**Authors:** Mohamad Bakir, Abdul Rahman Alkhatib, Abdul Rehman Mustafa, Mohammed Raddaoui, Wael Alkattan, Khalid Said Mohammad

**Affiliations:** 1 Department of Medicine, College of Medicine, Alfaisal University, Riyadh 11533, Saudi Arabia; mbakir@alfaisal.edu (M.B.); aralkhatib@alfaisal.edu (A.R.A.); abmustafa@alfaisal.edu (A.R.M.); mraddaoui@alfaisal.edu (M.R.); 2 Department of Surgery, College of Medicine, Alfaisal University, Riyadh 11533, Saudi Arabia; walkattan@alfaisal.edu; 3 Department of Anatomy, College of Medicine, Alfaisal University, Riyadh 11533, Saudi Arabia

**Keywords:** bone metastasis, nanoparticles, cancer immunotherapy, bone-targeted drug delivery, tumor microenvironment, stimuli-responsive nanocarriers

## Abstract

Bone metastases remain one of the most clinically devastating complications of advanced cancer, particularly in breast, prostate, and lung malignancies, where they drive pain, fractures, hypercalcemia, and progressive functional decline. Their management is further complicated by a highly immunosuppressive bone microenvironment characterized by osteoclast-driven bone destruction, myeloid cell dominance, impaired antigen presentation, and weak effector T-cell infiltration, all of which limit the activity of conventional immunotherapies. In this setting, nanoparticles are emerging not merely as passive drug carriers but as programmable platforms capable of reshaping the metastatic niche. This review discusses how bone-targeted and immune-responsive nanocarriers can improve therapeutic precision through hydroxyapatite-binding ligands, dual-targeting strategies, stealth coatings, enzyme- and pH-responsive release systems, and externally guided platforms. We further examine how these systems modulate key immune compartments within bone metastases, including reprogramming tumor-associated macrophages and myeloid-derived suppressor cells, restoring cytotoxic T-cell activity, enhancing dendritic-cell activation, and enabling in situ vaccination through photothermal or photodynamic immunogenic cell death. Particular attention is given to the delivery of checkpoint inhibitors, cytokines, siRNA/miRNA, mRNA, and clustered regularly interspaced short palindromic repeats (CRISPR)-based payloads, as well as to the rational combination of these with chemotherapy, bone-modifying agents, and radiotherapy. Finally, we highlight major translational barriers, including lesion heterogeneity, limited penetration into mineralized tissue, off-target immune effects, manufacturing complexity, and the continued lack of bone-specific preclinical and clinical validation. Collectively, immunomodulatory nanoparticles represent a promising strategy to convert bone metastases from immune-refractory sites into more therapeutically responsive lesions.

## 1. Introduction

The development of bone metastases is among the most severe outcomes in patients with advanced malignancies, commonly occurring in lung, breast, and prostate cancer [1,2]. These metastases lead to adverse clinical outcomes such as pathological fractures, hypercalcemia, and cancer cachexia [3], significantly impacting patients’ quality of life. Such outcomes are mediated by the bone tumor microenvironment, where myeloid cells such as osteoclasts, tumor-associated macrophages (TAMs), and myeloid-derived suppressor cells (MDSCs) promote a self-perpetuating vicious cycle through a variety of mechanisms that promote bone destruction, immune suppression, and tumor growth [4,5]. Osteoclasts drive bone resorption, which in turn releases growth factors and minerals stored in the bone matrix, directly stimulating tumor proliferation and survival [4,6,7,8,9]. Concurrently, TAMs and MDSCs establish a highly suppressive immune environment [10,11]. This is mediated through the suppression of cytotoxic T-cell activity, expansion of regulatory immune cell subsets, and secretion of suppressive cytokines. Furthermore, cytokines such as TGF-β, IL-10, and bone-derived immunosuppressive cues support this self-perpetuating metastatic niche by disabling antitumor immunity and reinforcing osteoclast-driven bone destruction. Collectively, these mechanisms impair T-cell infiltration and immune surveillance within the tumor microenvironment, leading to an “immune desert” phenotype [12]. Nanoparticle strategies are especially useful in bone metastasis because bone lesions present barriers that differ from those in soft-tissue tumors. Their mineralized structure, uneven vascular access, active osteoclast remodeling, and immunosuppressive marrow environment limit drug penetration and weaken antitumor immune responses. In this setting, nanocarriers can improve therapy by targeting hydroxyapatite-rich bone surfaces, releasing drugs in response to local cues such as acidity or proteases, and concentrating immunomodulatory payloads near tumor-adjacent bone.

Due to the immunosuppressive microenvironment of bones, immunotherapy faces several challenges in the management of bone metastases. Notably, the limited infiltration of T-cells likely explains the clinical finding that bone metastases are less responsive to checkpoint inhibitors than visceral metastases. Checkpoint inhibitors have revolutionized cancer therapy by enabling the body’s immune system to block inhibitory proteins, such as programmed cell death protein 1/programmed death-ligand 1 (PD-1/PD-L1), used by cancer cells [13]. This allows T-cells to effectively recognize and destroy cancer cells. While these inhibitors are generally better tolerated than chemotherapy and can improve the patient’s quality of life [14], their efficacy is reduced in bone metastases due to the highly immunosuppressive microenvironment, contributing to poorer clinical outcomes [15]. This clinical finding is largely attributed to the suppressive cytokines released during osteoclast-mediated bone resorption. TGF-β is one such cytokine released during this process, directly promoting immunosuppression by inhibiting the activation and trafficking of effector T-cells while driving the accumulation of regulatory T-cells (Tregs) [16].

Given the unique immunosuppressive microenvironment of bone metastases, nanocarriers hold great potential to enhance treatment efficacy and improve patient survival. This is achieved through various mechanisms, including nanoparticles’ ability to selectively target metastatic sites. This targeting not only enhances local therapeutic efficacy but also substantially reduces systemic toxicity [17,18], thereby limiting damage to healthy tissues and improving patients’ quality of life. In addition, nanocarriers can locally remodel the immunosuppressive bone microenvironment through various mechanisms, including alleviating tissue hypoxia, normalizing aberrant angiogenesis, inhibiting bone metastatic progression, and remodeling the extracellular matrix to facilitate drug and immune cell infiltration. They can also reprogram immune cell phenotypes, converting immunosuppressive cells into immunoactive populations through immune re-education. At the same time, nanocarriers help deliver multiple agents, further enhancing the ability to reshape the tumor microenvironment. This coordinated targeting of complementary pathways involved in immune suppression allows therapies to act synergistically within the metastatic niche. Nanocarriers also facilitate multi-agent therapeutic strategies by co-delivering cytokines and anticancer agents [19], promoting tumor regression while restoring local immune function. Moreover, their ability to deliver treatments directly at the tumor site and release them in a controlled manner can improve precision and help address resistance often observed with single-agent therapies.

**Figure 1:**
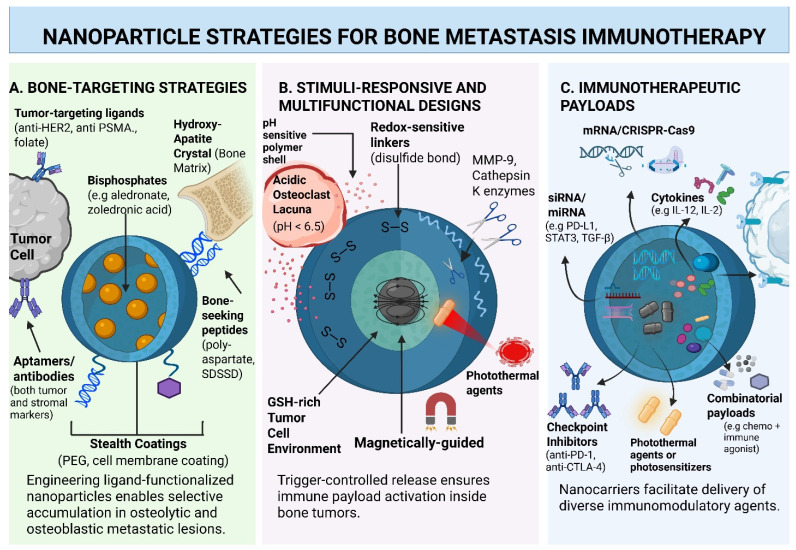
Nanoparticle design strategies for bone metastasis immunotherapy. (**A**) Bone-targeting strategies: nanocarriers can be directed to skeletal lesions by affinity for the bone mineral phase or through tumor-specific recognition, including bisphosphonates, hydroxyapatite-targeting interactions, bone-seeking peptides, tumor-targeting ligands, and antibody/aptamer-based approaches; stealth coatings are added to extend circulation and reduce nonspecific clearance. (**B**) Stimuli-responsive and multifunctional designs: nanoparticles can be engineered to respond to key features of the bone metastatic environment, such as acidic osteoclast lacunae, glutathione-rich tumor cells, and protease-rich sites like MMP-9- or cathepsin K-enriched lesions, while also incorporating photothermal modules or magnetic guidance for spatially controlled activation. (**C**) Immunotherapeutic payloads: typical cargos include siRNA/miRNA, mRNA, and CRISPR-Cas9 systems, cytokines, checkpoint inhibitors, photothermal or photosensitizing agents, and combination payloads designed to suppress tumor growth while restoring antitumor immunity. These engineering features enable targeted skeletal accumulation, controlled intralesional release, and local immune modulation in bone metastases. Created in BioRender. Mohammad, K. (2026) https://BioRender.com/xe0pbil (accessed on 22 April 2026).

The aim of this review is to examine the emerging role of immunomodulatory nanoparticles in treating bone metastases. Beyond serving as carriers for drug therapies, these nanocarriers actively reprogram the immunosuppressive nature of the tumor microenvironment, utilize combination and gene therapies, and enhance anti-tumor immune responses to suppress tumor progression. Building on these capabilities, this review also seeks to identify critical research gaps in how immunomodulatory nanoparticles interact with the bone tumor microenvironment and define key translational barriers that must be addressed to advance nano-immunotherapies for bone metastases. The principal nanoparticle design strategies and immunotherapeutic payload classes discussed in this review are summarized in Figure 1A–C.

## 2. Engineering Strategies for Bone-Targeted Immunomodulatory Nanoparticles

### 2.1. Design Principles and Targeting Moieties for Bone-Directed Nanoparticles 

#### 2.1.1. Taxonomy and Functional Roles of Nanoparticle Modalities

In the context of bone metastasis immunotherapy, the most relevant nanoparticle modalities are lipid/liposomal systems, polymeric nanoparticles, bone-targeted ligand-decorated platforms, inorganic photothermal or magnetic nanoparticles, and biomimetic membrane-coated or exosome-like systems [20]. These classes are not interchangeable. Lipid and polymeric systems are generally the most versatile for loading nucleic acids, cytokines, and combination payloads, whereas ligand-decorated systems are most directly suited for skeletal localization. Inorganic platforms are particularly relevant when externally triggered functions such as photothermal ablation, magnetic guidance, or theranostic imaging are desired [21]. Biomimetic systems may improve circulation, immune evasion, and lesion interfacing, but currently remain less standardized for translation [22]. Accordingly, “nanoparticles” should not be treated as a single modality, but rather as a set of distinct engineering classes with different biological roles and translational constraints.

#### 2.1.2. Bone-Targeting Ligands

Bisphosphonates (BPs), including alendronate (ALN) and zoledronate (ZOL), are extensively utilized as bone-targeting ligands on nanoparticles due to their robust, multidentate affinity for calcium in hydroxyapatite (HAp), the principal mineral component of bone. This same chemistry underpins their traditional anti-resorptive function and also influences the location and mechanism of action of BP-decorated immunomodulatory nanocarriers within the bone microenvironment. Bisphosphonates feature a P–C–P backbone that chelates Ca^2+^ in hydroxyapatite (HAp); 1-hydroxy-1,1-bisphosphonates and nitrogen-containing BPs (e.g., alendronate, zoledronate) bind strongly and frequently irreversibly to bone mineral surfaces [23,24,25,26].

ZOL exhibits the highest affinity for calcium phosphate, succeeded by ALN and other bisphosphonates, establishing ZOL and ALN as the predominant bone-targeting ligands in nanodelivery systems [27]. The ZOL or ALN alteration enhanced nanoparticle accumulation in bone by approximately 50-fold and 8-fold, respectively, compared to non-bisphosphonate controls [27]. ALN-decorated PLGA, liposomal, and polymeric systems consistently exhibit superior binding to HAp disks/powders compared to non-modified carriers, resulting in increased accumulation in bone or bone tumors in vivo [23,25,27,28]. ALN-decorated iron oxide nanoparticles (IONPs) and magnetite nanoparticles demonstrate significant bone accumulation following systemic administration [27,29,30] (Figure 1A).

Larger self-assembling bisphosphonate-decorated nanoparticles.

Nanoparticles (>100 nm) typically exhibit enhanced in vitro hydroxyapatite binding compared to smaller counterparts, presumably due to a greater number of bisphosphonate-decorated nanoparticle groups interacting with mineral surfaces [23]. Ultimately, there exists an optimal nanoparticle concentration for maximal hydroxyapatite binding; high nanoparticle concentrations diminish the binding percentage, perhaps due to surface saturation and nanoparticle-nanoparticle interactions [23].

Calcium bisphosphonate nanoparticles infiltrate extensively and distribute uniformly inside human bone and cartilage explants while maintaining a strong mineral affinity. BP-modified hydrogels and nanocomposites utilize BP–nHA chelation to generate thick, evenly mineral-anchored networks that facilitate bone regeneration [24,31]. BP–HAp interactions concentrate nanoparticles at resorbing surfaces and mineral-dense areas, particularly trabecular surfaces and fracture/defect sites, where hydroxyapatite exposure and micro-damage are prevalent [24,26,27].

There is substantial evidence that BP-targeted nanosystems influence immune cells in bone, specifically macrophages and osteoclast lineage cells. Macrophage targeting and repolarization via ZOL formulations, including calcium–zoledronate nanoparticles embedded in microparticles (CaZol NiM), facilitate sustained ZOL release to macrophages, diminish direct cytotoxicity, inhibit nuclear factor kappa B (NF-κB) and ROS activity, encourage M1 to M2 repolarization, and expand the applicability of ZOL for both skeletal and extra-skeletal inflammatory diseases [32]. While this platform is not directly anchored to bone by mineral binding, other Ca–BP constructions exhibit significant mineral affinity and bone penetration [27,33], suggesting that bone-targeted, BP nanoparticle (NP)-mediated macrophage immunomodulation is plausible.

Aminobisphosphonates, particularly ZOL, exhibit well-characterized immunomodulatory effects on macrophages and γδ T-cells, resulting in cytokine release and anti-tumor immune responses [34]. When employed as targeting ligands on nanocarriers, these medicines can concurrently deliver an immunomodulatory payload and exert intrinsic BP-mediated immunological effects at osseous locations [34].

In the design of immunomodulatory (BP) nanoparticles, such as those intended for mRNA vaccines, cytokine modulators, or immune adjuvants, it is advisable to utilize high-affinity BPs (ZOL ≥ ALN) when robust mineral anchoring is required. However, it is important to recognize that overly strong binding may restrict penetration into tumor cores and preferentially promote localization at bone surfaces. Certain systems employ detachable or dual-targeting motifs to achieve a balance between bone and tumor localization [23,35]. Furthermore, it is crucial to adjust BP surface density and particle size to enhance HAp binding while maintaining circulation time, biodistribution, and tissue penetration [23,27]. Researchers can leverage dual functionality, as BPs can work as both targeted ligands and active pharmaceuticals, integrating anti-resorptive and anti-tumor or immunomodulatory effects with the nanoparticle’s payload [27,34]. Ultimately, one can exploit local immune modulation by incorporating reactive oxygen species (ROS) scavenging cores (such as iron oxide and antioxidants) alongside ALN to adjust redox and immune conditions at bone surfaces, or by employing ZOL-based or other nitrogen-containing bisphosphonate (N-BP) platforms to target and repolarize macrophages and osteoclasts, or to synergize with immune adjuvants or mRNA therapies.

Recent research indicates that BP-based targeting effectively directs nanoparticles to calcified bone and influences local osteoclasts, bone marrow stromal cells (BMSCs), reactive oxygen species (ROS), and macrophages, frequently reinstating a more anabolic, pro-regenerative milieu [27,29,31,32,36,37]. The temporal dynamics of these effects concerning typical “anabolic windows” remain inadequately characterized, and direct comparisons between BP-NP and non-BP-NP immunotherapies regarding long-term bone formation/resorption coupling are limited. To address these gaps, longitudinal in vivo investigations must be conducted to monitor bone formation and resorption markers, immune cell morphologies, and the spatial distribution of BP-NPs across trabecular, cortical, and marrow compartments.

#### 2.1.3. Bone-Targeting Peptides

Bone metastases originating from breast and prostate cancer exhibit upregulation of αvβ3 and αvβ5 on tumor cells, osteoclasts, and neovessels, enhancing adherence to the bone extracellular matrix. Arginine-glycine-aspartate (RGD) binding facilitates the attachment of cancer cells to bone and is associated with the selective migration of specific malignancies to skeletal locations [38]. Clinical positron emission tomography (PET) findings suggest early elevation of αvβ3 in bone microenvironments during metastasis, with RGD tracer uptake in bone lesions akin to FDG (Fluorodeoxyglucose), indicating integrin expression on metastatic cells, endothelial cells, and osteoclasts, rather than solely on tumor cells [39]. RGD peptides and their derivatives, especially iRGD, exhibit a high affinity for αvβ3 and αvβ5, which are closely linked to tumor angiogenesis, invasion, and metastatic spread. In bone-metastatic breast cancer, the overexpression of αvβ3 on bone-tropic cells is identified as a significant target for drug delivery [40].

In an immunocompetent model, RGD-functionalized PEG-gold nanoparticles exhibited improved in vitro absorption in cancer cells, yet demonstrated diminished in vivo tumor formation owing to heightened identification and clearance by the mononuclear phagocyte system, resulting in greater uptake by the liver and spleen [41]. This highlights that unprotected RGD can diminish circulation and decrease net delivery, and that immunological state significantly affects biodistribution [41,42].

αvβ3 and αvβ5 are pivotal regulators of tumor angiogenesis, exhibiting significant expression on neovascular endothelium and some tumor cells, hence facilitating endothelial migration, survival, and vessel sprouting. RGD-binding ligands and disintegrins can obstruct αvβ3/αvβ5, consequently preventing endothelial cell invasion, tube formation, and neovessel maturation, which results in a decreased microvessel density in malignancies and bone metastases [43,44,45]. A liposomal RGD-disintegrin (vicrostatin) markedly reduced tumor-associated microvessel density in prostate cancer xenografts and effectively inhibited the growth of bone metastases, aligning with its anti-angiogenic properties and integrin blocking.

#### 2.1.4. Antibodies and Aptamers

Aptamer-functionalized nanoparticles demonstrate efficient prostate-specific membrane antigen (PSMA)-mediated delivery and in vivo tumor suppression in bone metastasis models with minimal immunogenicity, whereas antibody-based strategies (including bone-homing engineered antibodies) exhibit robust HER2 targeting but necessitate bone-targeting modifications to address inadequate bone access; both approaches are still in the preclinical stage and face significant challenges from the immunosuppressive bone marrow microenvironment. Aptamer-anchored HPAA specifically targeted PSMA on prostate cancer cells, facilitating cellular uptake and the delivery of miRNA cargo to PSMA-expressing LNCaP cells in vitro and to tibial tumors in vivo. Engineering antibodies with bone-homing peptide sequences or conjugating bone-targeting moieties enhanced antibody concentration within bone metastatic niches and augmented therapeutic efficacy in preclinical animals [46]. Systemically administered nanoparticles containing stimulator of interferon genes (STING) agonists localized in the bone marrow and persisted in tumor microenvironments for over 24 h, indicating that nanoparticle platforms can facilitate sustained bone marrow delivery when properly engineered [47].

Aptamers exhibit minimal immunogenicity, a beneficial characteristic for repeated systemic delivery and for mitigating off-target immune activation [48]. The HPAA-PEG-APT/miRNA complex exhibited reduced cytotoxicity and enhanced biocompatibility in vitro [47]. Therapeutic antibodies are widely used in clinical settings; nevertheless, the literature highlights constraints related to size and dispersion, rather than assessing the immunogenicity of bone-targeted antibody complexes. Large biologics exhibit increased manufacturing complexity and a heightened risk of anti-drug immune reactions [47,48]. Nanoparticle cargo can elicit immunological responses; for instance, STING-agonist nanoparticles stimulated proinflammatory cytokine production and initiated early T-cell activation [47], while antibody-functionalized photodynamic nanoparticles induced immunogenic cell death and activated dendritic, T, and natural killer (NK) cells [49]. Bone marrow immune suppression limits therapy; many nanoparticle techniques have demonstrated the ability to modulate the bone marrow microenvironment in various ways. Systemically administered polymer nanoparticles containing cyclic dinucleotide STING agonists elicited proinflammatory cytokine production but subsequently caused immunosuppressive responses (increased immunosuppressive cytokines and Treg infiltration), hence limiting efficacy after about two weeks [47].

Aptamers face challenges such as stability in circulation, vulnerability to nucleases, and the necessity to exhibit robust in vivo pharmacokinetics and scalability in production [48]. Antibodies such as trastuzumab exhibit confirmed clinical efficacy and can be reused with bone-targeting motifs; nevertheless, they encounter limitations in tissue penetration [47]. Existing evidence for both is preclinical: HPAA-PEG-APT, designed bone-homing trastuzumab, and bone-targeted antibody–drug conjugate (ADC) designs have shown efficacy in murine models but have not been documented in clinical trials [47,48].

### 2.2. Multifunctional Surface Engineering

#### 2.2.1. Dual-Targeting Systems

Dual-targeted systems for bone metastasis leverage two distinct levels of specificity, employing a bone-homing ligand, such as alendronate, to facilitate accumulation in the mineralized metastatic environment, and a tumor-specific ligand, such as folate, phage peptide, cell-membrane coating, or RGD/PSMA analogs, to selectively attach to malignant cells within that environment. This tiered design is openly realized in many bone metastasis platforms and theoretically reflected in cascade-targeting nanomedicines (Figure 1B).

Alendronate (ALN) and other bisphosphonates exhibit a great affinity for hydroxyapatite, resulting in significant bone-targeting affinity of nanoparticles and their accumulation in areas of elevated bone turnover, which are often sites of metastasis. ALN-decorated micelles or PLGA nanoparticles accumulate 10–20 times higher in bone tumor settings than in healthy bone due to tumor-induced osteolysis, which exposes new minerals and concentrates bisphosphonates near the lesions [50]. Ultimately, ALN-conjugated shells on zeolitic imidazolate framework-8 (ZIF-8) nanoparticles or hyaluronate nanoparticles similarly enhance deposition in metastatic skeletal locations in comparison to non-ALN controls [51,52]. This enhances the local drug concentration at bone lesions while minimizing exposure to non-skeletal organs, hence enhancing the therapeutic index compared to free drugs [50,53,54].

In bone, the majority of cells are non-malignant (osteoblasts, osteoclasts, stromal cells, hematopoietic stem and progenitor cells, lymphoid/myeloid cells); nevertheless, nested targeting introduces an additional recognition layer to preferentially enhance uptake by tumor cells. Targeting folate receptors with ALN/folic acid-decorated PLGA nanoparticles facilitates the delivery of paclitaxel to breast cancer cells that overexpress folate receptors following initial localization in the bone. This approach significantly enhances intralesional accumulation, suppresses tumor growth and lung metastasis, and diminishes toxicity to normal tissues compared with non-targeted paclitaxel [55]. Moreover, the tumor-binding phage peptide (DP 8) within ALN/DP 8 micelles is engineered to ensure ALN targets osteoclast-rich metastatic bone, while DP 8, which binds to surface nucleolin, enhances micelle concentration on breast cancer cells. This strategy mitigates ALN’s propensity to sequester nanoparticles on osteoclasts, thereby augmenting antitumor efficacy with minimal systemic toxicity [50]. Similarly, the application of homotypic tumor membrane coating utilizing ALN–hydroxyapatite targeting, in conjunction with multiple myeloma cell membrane on BTZ-loaded PLGA vesicles, facilitates a “bone-first, MM-cell-next” delivery mechanism. This approach leverages MM membrane adhesion molecules to enhance specific recognition and uptake by myeloma cells within the marrow, thereby augmenting intralesional bortezomib retention, intensifying proteasome inhibition, and mitigating systemic toxicity [56]. Ultimately, non-bone instances, such as the CD44-then-mitochondria cascade or PD-1 targeted T-cell nanoparticles, demonstrate that sequential targeting layers systematically enhance cellular selectivity and intratumoral drug concentration compared to single targets or free drugs [57,58,59].

Bone-targeted GANT58 BTNPs enhance drug concentrations in tumor-associated bone, markedly diminish bone degradation, and augment bone volume with minimum systemic toxicity, surpassing both untargeted nanoparticles and drug-free ALN nanoparticles [53]. Moreover, dual ALN/folate or ALN/DP 8 complexes provide enhanced tumor control and survival advantages compared to single ligands or free drugs, while concurrently mitigating unfavorable effects on normal tissues [50,55].

Healthy bone marrow immune cells, such as hematopoietic stem and progenitor cells (HSPCs), T and B lymphocytes, macrophages, and dendritic cells, are endangered due to their propensity to phagocytose particles, serving as crucial mediators of systemic toxicity and immunosuppression [60,61]. Nested targeting safeguards these cells via spatial confinement, since ALN localizes nanoparticles at metastatic sites characterized by elevated bone turnover and osteoclast activity, whereas healthy bone with diminished turnover accumulates much fewer ALN-laden nanoparticles [50,53]. This protection is augmented by receptor limitation, wherein additional ligands like folate, nucleolin-binding DP 8, or tumor membranes interact with receptors that are overexpressed on cancer cells but are either low or missing on the majority of healthy marrow subsets [50,55,56]. Functional separation is evidenced by GANT58 NP therapy, which selectively diminishes tumor-induced osteoclast activation while preserving physiological osteoclast function in non-tumor locations, suggesting that drug exposure and/or sensitivity is markedly elevated in the tumor-conditioned niche compared to normal bone [53].

Reviews on immune cell-targeted nanoparticles highlight that multi-ligand or enzyme-responsive designs can direct particles to specific immune subsets while minimizing bystander uptake; in bone metastasis [59,62]. This design principle seeks to evade hematopoietic stem and progenitor cells and benign myeloid cells unless they are intended therapeutic targets.

#### 2.2.2. Stealth Coatings

PEGylation creates a hydrated steric barrier that prevents opsonin adsorption and subsequent uptake by the Mononuclear Phagocyte System (MPS), hence prolonging systemic half-life and enhancing tissue distribution [63,64,65]. The effectiveness of this ‘stealth’ effect is determined by various design trade-offs: factors such as molecular weight, surface density, and polymer architecture (e.g., linear versus branched) influence the shift between ‘mushroom’ and ‘brush’ conformations. High-density ‘brush’ PEG optimally inhibits phagocytosis and extends circulation time, but it may concurrently obstruct target-cell internalization [64,65,66,67]. Research on SN38 nanoprodrug assemblies indicated that minimal PEGylation (≤20%) resulted in fast clearance by the mononuclear phagocyte system, while highly PEGylated (150% *w*/*w*) nanoparticles exhibited much extended circulation and maximal tumor formation, with no observable anti-PEG response in that model [67]. Comparable patterns are observed in various PEGylated systems; however, certain pH-sensitive liposomes exhibited no advantages in circulation or tumor uptake due to PEG, highlighting formulation dependence [68].

Cell membrane coatings mimic entire cell antigenic and signaling environments on synthetic cores, merging stealth with active targeting. Red blood cell (RBC) membrane-coated nanoparticles utilize CD47-SIRPα signaling to suppress macrophage phagocytosis, resulting in prolonged circulation without antibody-mediated clearance following repeated administration and minimal stimulation of humoral and cellular immune responses [69]. Leukocyte and macrophage membranes incorporate chemokine receptors and adhesion molecules that facilitate evasion of the mononuclear phagocyte system and target inflamed or neoplastic regions [70,71,72]. Cancer cell membranes provide homotypic adhesion and immune evasion characteristics, facilitating tumor formation and prolonged circulation relative to uncoated nanoparticles [63,70,73,74]. Comparative analysis of polystyrene nanoparticles revealed that breast cancer cell membrane-coated nanoparticles had a reduced protein corona formation and enhanced tumor cell uptake compared to PEG-coated particles, while simultaneously minimizing nonspecific protein adsorption [73].

The MPS organs (liver, spleen, and marrow macrophages) serve as key sinks for nanoparticles; opsonization facilitates their uptake by Kupffer cells and splenic/red pulp macrophages [64,65]. PEG diminishes opsonin adsorption and phagocytosis, thereby reducing MPS organ accumulation; however, splenic uptake may remain significant and can even be augmented based on PEG architecture (e.g., branching PEG enhances leukocyte-mediated splenic accumulation) [64,65,75]. Glycopolymer coatings diminish the adsorption of immunogenic proteins (immunoglobulins, complement) and exhibit reduced liver uptake and enhanced tumor-to-liver ratios compared to PEGylated counterparts, indicating that more “biological” hydrophilic shells may surpass PEG in reducing MPS capture [76].

High-density PEG is well-validated for pure circulation extension and generic MPS avoidance; nonetheless, it requires meticulous adjustment to prevent ABC, immunogenicity, and reduced cellular absorption [64,65,66,67,77,78]. In the context of repeated dosing, reduced immunogenicity, and immune checkpoint-mediated evasion, RBC or leukocyte membranes demonstrate superiority over traditional PEGylation, particularly in preclinical models [69,70,71,72,79].

Endogenous stimulus-responsive nanoparticles have emerged as a viable approach to address the distinct physiological barriers of the bone metastatic niche and to facilitate accurate, site-specific medication administration. These intelligent nanocarriers utilize localized microenvironmental signals such as acidic pH, hypoxia, and overexpressed enzymes (e.g., MMP-9, Cathepsin K) to initiate regulated drug release specifically at metastatic bone lesions. pH-sensitive linkers (particularly hydrazone bonds), hypoxia-responsive elements (such as azobenzene derivatives), and enzyme-cleavable substrates are fundamental to this strategy, enabling targeted payload release while reducing systemic toxicity. Recent advancements indicate that these methods can augment drug accumulation in bone metastases, boost treatment efficacy, and diminish off-target effects by utilizing the unique metabolic environment of bone cancers [80,81,82,83,84,85,86]. Nonetheless, obstacles persist regarding the heterogeneity of the tumor microenvironment, the depth of penetration into dense osseous tissue, and clinical applicability.

The acidic environment typical of bone metastases (pH ~ 6.5–6.9) is extensively utilized through pH-sensitive linkers, including hydrazone bonds [80,81,82,83,87,88,89,90,91]. These linkers exhibit stability at normal pH but undergo hydrolysis in the acidic environments characteristic of malignancies or intracellular compartments (endosomes/lysosomes), hence initiating drug release preferentially in metastatic locations [82,88]. Hydrazone-linked doxorubicin prodrugs exhibit fast release at pH 5–6, while demonstrating negligible leakage at neutral pH [80,87]. Systems that are dual-sensitive, integrating pH-responsiveness with redox or enzymatic sensitivity, significantly improve selectivity [82].

Hypoxic areas within bone metastases stimulate nanocarriers that incorporate hypoxia-sensitive components, like azobenzene or nitroimidazole derivatives [80,83,84,92,93]. Azobenzene linkers are subject to reductive breakage by tumor-associated azoreductases in hypoxic settings, hence releasing active pharmaceuticals only inside hypoxic tumor regions [80,83]. These approaches have exhibited increased cytotoxicity towards hypoxic cancer cells while preserving normoxic tissues [92].

Bone-specific enzymes such as matrix metalloproteinase-9 (MMP-9) and Cathepsin K are overexpressed in metastatic lesions and osteolytic settings. Nanoparticles modified with peptide substrates susceptible to enzymatic cleavage facilitate precise drug release upon interaction with their target enzymes [81]. MMP-cleavable linkers integrated into polymeric micelles or dendrimers facilitate fast payload release solely in enzyme-dense tumor environments [81,94].

The dense extracellular matrix (ECM), elevated interstitial fluid pressure, and restricted vascularization provide considerable obstacles to nanoparticle infiltration in bone metastases [81,85,95]. Collagenase-loaded nanocapsules released in acidic environments have demonstrated the ability to locally break down extracellular matrix components and enhance nanoparticle diffusion into deep tumor areas [95]. Surface alterations utilizing bisphosphonates or peptides that target hydroxyapatite significantly augment accumulation in bone tissue [82].

### 2.3. Bone Lesion Architecture and Design Implications: Osteolytic Versus Osteoblastic Metastases

Different primary tumor origins have different architectures, which further complicates these barriers. For example, breast cancer typically produces osteolytic lesions that have a chaotic vascular supply and an acidic microenvironment. Prostate cancer, in contrast, produces osteoblastic lesions driven by factors like endothelin-1 (ET-1) and bone morphogenetic proteins (BMPs). As a result, the vessels in these dense, sclerotic lesions are poorly permeable, restricting the entry of nanotherapeutics [96]. This difference between osteolytic and sclerotic lesions is confirmed by imaging, such as 18F-sodium fluoride PET/CT [97]. The architectural diversity of bone metastases establishes unique physiological barriers that determine the effectiveness of nanoparticle delivery. In osteolytic lesions, the aberrant enhancement of osteoclast activity and ensuing bone resorption compromise the skeletal architecture, resulting in resorption lacunae and elevated receptor activator of nuclear factor kappa-B ligand (RANKL) signaling; notwithstanding these structural alterations, these regions are comparatively poorly perfused, limiting the accessibility of systemically administered therapeutic agents [35,98,99,100,101]. Conversely, osteoblastic lesions exhibit a dense, hypertrophied extracellular matrix and the accumulation of new bone, which obstructs drug permeation and restricts accumulation at the metastatic site, consequently diminishing uptake by cancer and stromal cells. The inherent bone–bone marrow barrier and the typically reduced blood flow in the osseous niche impede NP accumulation in both lesion types, in contrast to the more advantageous hemodynamics of soft tissues [35,40,99]. Neutral, approximately 150 nm sized nanoparticles localized to bone marrow and metastatic sites approximately seven-fold more effectively than 320 nm sized or charged nanoparticles in an osteolytic prostate model, enhancing co-localization with tumors in the marrow and mitigating bone loss [40,99].

Current preclinical research predominantly employs osteolytic breast and prostate models; explicit comparisons of osteoblastic or mixed models regarding NP penetration are lacking [35,40,98,99,100,101]. Reviews indicate diminished drug permeation in osteoblastic metastases attributed to a dense matrix, yet lack quantitative data on nanoparticle delivery compared to osteolytic lesions [40]. Moreover, one meta-analysis of solid tumors indicates a generally low tumor nanoparticle delivery rate (~1–2% of the injected dose), underscoring that microenvironmental permeability, rather than solely targeting ligands, constitutes a significant limitation [102]. 

**Figure 2:**
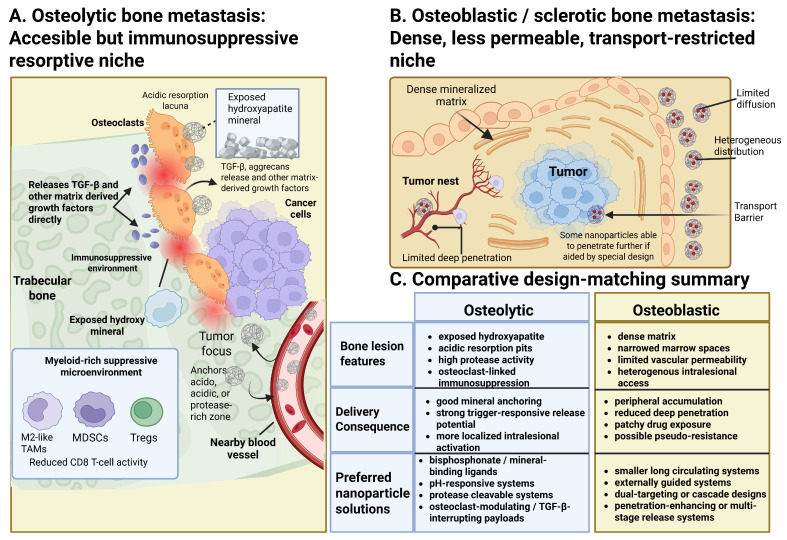
Lesion architecture-guided design considerations for nanoparticle delivery in osteolytic versus osteoblastic bone metastases. (**A**) Osteolytic bone metastasis forms a relatively accessible but profoundly immunosuppressive resorptive niche characterized by osteoclast-rich bone erosion, acidic resorption lacunae, exposed hydroxyapatite surfaces, protease-rich microdomains, release of TGF-β, and other matrix-derived factors, as well as a myeloid-dominant suppressive microenvironment that limits effective antitumor immunity. These features can favor mineral anchoring and locally triggered intralesional release, but they also reinforce tumor progression and immune dysfunction. (**B**) Osteoblastic/sclerotic bone metastasis forms a dense, poorly permeable, transport-restricted niche marked by newly mineralized matrix, narrowed marrow spaces, limited vascular permeability, reduced diffusion, and heterogeneous intralesional distribution, resulting in shallow penetration and patchy drug exposure. (**C**) Comparative design-matching summary of the two lesion types. Osteolytic lesions are generally more compatible with mineral-binding ligands, pH-responsive systems, protease-cleavable platforms, and osteoclast- or TGF-β-modulating payloads, whereas osteoblastic lesions may require smaller long-circulating nanoparticles, externally guided systems, dual-targeting or cascade strategies, and penetration-enhancing or multistage-release designs. Overall, the figure emphasizes that lesion architecture and osteoimmune context should guide nanoparticle design, delivery logic, and payload selection in bone metastasis. Created in BioRender. Mohammad, K. (2026) https://BioRender.com/nd4d769 (accessed on 22 April 2026).

Research indicates that in osteolytic bone metastasis models, meticulous adjustment of nanoparticle size and charge, bone-targeting ligands, and microenvironment-modulating cargos can enhance marrow localization, co-localization with tumors, and inhibition of osteolysis. Osteoblastic lesions are characterized by a denser matrix and reduced permeation; however, there is a lack of direct, quantitative comparisons of nanoparticle intratumoral penetration between osteolytic and osteoblastic bone metastasis models. These lesion-specific architectural differences, their delivery consequences, and the corresponding nanoparticle design strategies are summarized in Figure 2.

### 2.4. Enzyme-Responsive Nanocarriers

Enzyme-responsive nanocarriers utilize locally overexpressed proteases and phosphatases in bone metastases to transition from a “stealth/transport” state to a “active/releasing” state, enhancing both accuracy and efficacy compared to non-responsive nanoparticles.

The literature illustrates that MMP-9, CTSK, and ALP serve as optimal triggers in bone metastasis due to the distinctive enzyme dysregulation observed in bone metastatic habitats. Matrix metalloproteinases, particularly MMP-2/9, are increased in invasive, metastatic cancers and remodel bone and marrow extracellular matrix, associated with unfavorable prognosis and metastasis development [103,104]. Moreover, Cathepsin K (CTSK) is significantly overexpressed in osteoclasts and bone metastatic microenvironments, and as CTSK levels in bone lesions surpass those in original tumors or soft tissue metastases, it functions as a bone-selective metastasis marker and initiator [105]. Alkaline phosphatase (ALP), especially tissue-nonspecific or bone ALP, is enhanced in osteoblastic remodeling and metastatic bone disease. Thus, ALP-cleavable groups are extensively utilized to reveal charges or form insoluble deposits in mineralizing and bone-like contexts; yet, comprehensive studies on ALP-responsive systems in bone metastasis are less prevalent than those on MMP/CTSK in the literature. These enzymes are primarily confined to pathological environments, offering biochemical specificity that surpasses the mere passive Enhanced Permeability and Retention effect or bone affinity alone [40,106,107].

Engineered enzyme triggers regulate cargo release via various intricate methods, especially in MMP-9-responsive systems. Fundamental design ideas derived from cancer models, applicable to bone metastasis, include the incorporation of cleavable peptide linkers (e.g., PLGLAG) between PEG shields and cationic/drug-loaded cores, or between gating caps such as avidin or peptides and mesoporous silica pores [103,104,108]. Prior to cleavage, these nanoparticles are generally PEGylated, neutral, or negatively charged, which guarantees their stability in the bloodstream and minimizes premature release [103]. Upon exposure to MMP-9 in tumor or bone lesions, these peptide linkers undergo hydrolysis, resulting in the detachment of PEG or caps and the exposure of cationic surfaces or open pores. This change results in improved cellular absorption through unmasked positive charges or ligands and promotes fast drug diffusion from the expanded pores [103,104,108]. Particular processes involve the elimination of MMP-9-cleavable PEG shells to enhance the absorption of siRNA/PTX micelles, thus augmenting tumor inhibition and metastasis reduction [103,104]. MMP-9 can transform peptide micelles into nanofiber depots that localize doxorubicin, facilitating a gradual, localized release that effectively inhibits tumor development [109,110]. In a design unique to bone metastases, MMP-sensitive regions may be integrated with bone-binding ligands.

Enzyme-responsive carriers enhance results relative to non-responsive nanoparticles by providing greater spatial accuracy and micro-scale targeting. Moreover, these carriers markedly diminish systemic toxicity and off-target harm. MMP-9-responsive MSNs release cisplatin exclusively in MMP-9-elevated lung tumor areas, thereby preserving normal lung tissue. This principle, when applied to bone metastasis, could localize cytotoxic agents to enzyme-rich bone lesions and minimize marrow damage more effectively than continuous-release nanoparticles. This is facilitated by improved cellular uptake and intratumoral retention, as enzyme-mediated de-PEGylation or charge reversal resolves the dilemma between prolonged circulation and cellular entry; PEG shielding and negative charges are preserved during transport and modified solely within the lesion [103,104,105].

Ultimately, these systems utilize pathology-specific enzyme profiles, exemplified by the markedly elevated levels of CTSK in bone metastases relative to primary tumors, facilitating differentiation across various tumor sites [105]. Thus, protease-activated systems facilitate multi-site selectivity, activating solely in enzyme-abundant lesions [103,104,111]. By aligning the trigger—MMP-9, CTSK, or ALP—with the enzymatic profile of bone metastases, researchers acquire a therapeutic index that conventional nanoparticles cannot achieve.

### 2.5. Physically Guided Targeting

#### 2.5.1. Magnetic Nanoparticles

SPIONs offer a mechanism for magnetically concentrating therapy in bone tumors and creating heat for synergistic chemo-hyperthermia, although direct evidence in poorly vascularized bone metastases is scarce.

Superparamagnetic iron oxide nanoparticles (SPIONs) can be directed and held at pathological areas with the use of external magnetic fields (“magnetic targeting”), hence enhancing local nanoparticle accumulation beyond what is attainable through the passive enhanced permeability and retention (EPR) effect alone [112,113,114,115]. Aggregating SPIONs into multicore assemblies or nanoclusters enhances magnetic susceptibility, hence augmenting responsiveness to external fields and enhancing site-specific retention under therapeutically relevant field strengths [112,113,114,116].

In bone cancer, the vascular supply is frequently diverse and partially necrotic, which restricts systemic medication penetration. Magnetically guided systems can theoretically mitigate low perfusion through field-driven margination and retention, wherein SPIONs are drawn from circulation into the peritumoral microvasculature and retained against washout, thereby enhancing residence time and facilitating extravasation even in areas with sluggish flow [112,113,114,115]. Additionally, magnetically actuated scaffolds and gels can be employed in bone applications; for instance, injectable PLGA gels co-loaded with Fe_3_O_4_/MgCO_3_ create a localized magnetic depot in bone malignancies, with SPIONs kept in situ and stimulated by alternating magnetic fields (AMF) for many treatments [117,118]. The application of static or rotating magnetic fields can augment cellular absorption by elevating the intracellular concentration of SPIONs in tumor cells and 3D microtissues, therefore reducing the duration of hyperthermia and decreasing the required particle dosage [119]. While these investigations concentrate on osteosarcoma or soft tissue malignancies instead of traditional bone metastases, they illustrate that external fields might mitigate inadequate vascularization by mechanically entrapping SPIONs or facilitating their deeper penetration into tissue [117,118,119,120]. External fields enhance the capture of SPIONs from circulation near skeletal lesions, hence mitigating poor perfusion and reducing clearance. Rotating magnetic fields inhibit chain-like aggregation and facilitate deconstruction into units that are more effectively swallowed by 3D microtissues [119], whereas magnetic hyperthermia enhances transport across dense, mineralized matrices [121].

Despite these advancements, the majority of data originate from primary osteosarcoma or soft tissue models rather than traditional breast, prostate, or lung bone metastases [117,118,120,122]. There is a deficiency of direct comparison research on SPION deposition in weakly vascularized bone metastases, as the evidence predominantly depends on general tumor models. Moreover, optimizing field geometry for deep skeletal targets, such as the spine or pelvis, continues to pose an engineering challenge, and clinical trials for these particular applications are still scarce.

SPION-mediated magnetic hyperthermia (MHT) can elicit immunogenic cell death (ICD) and modify tumor immunity, establishing a mechanistic foundation for “in situ vaccination” that may complement immune checkpoint inhibitors, although direct evidence in bone metastases is scarce and primarily derived from other solid tumors. In skeletal disease, this concept should currently be regarded as mechanistically plausible and partially supported, rather than broadly validated, because direct bone metastasis evidence remains limited. SPIONs transform alternating magnetic field (AMF) energy into thermal energy, facilitating targeted intratumoral temperatures between 39 and 45 °C while preserving adjacent tissues [112,123]. In vivo SPION MHT devices can sustain tumor temperatures about 45 °C, leading to considerable growth inhibition and localized heat shock reactions [112,123,124,125]. Mild MHT below 44–45 °C is increasingly preferred to prevent antigen degradation observed at ablative temperatures over 50 °C and to enhance immunogenicity rather than solely inducing coagulative necrosis [112,126,127]. In osseous and other deep locations, the primary benefits of SPION MHT include extensive field penetration and localized heating at nanoparticle accumulation sites, crucial for addressing metastatic foci within mineralized bone [112,128]. Nonetheless, specialized preclinical models for SPION MHT in bone metastasis remain limited; most mechanistic data are derived from subcutaneous or cerebral malignancies and are extrapolated to bone.

Superparamagnetic iron oxide-based magnetic hyperthermia (MHT) targeting mitochondrial heat stress significantly elevated mitochondrial ROS, resulting in endoplasmic reticulum (ER) stress and the production of calreticulin (CRT), adenosine triphosphate (ATP), and heat shock protein 70 (HSP70) as indicators of immunogenic cell death (ICD), which reprogrammed M2 macrophages to adopt anti-tumor M1 phenotypes [127]. SPION MHT reviews highlight that hyperthermia may induce ICD-like cell death rather than just necrosis, accompanied by DAMP release and subsequent antigen presentation [112,124,127,128]. A study on in vivo SPION MHT revealed localized elevation of HSP70 in heated tumors, accompanied by tumor suppression and an influx of activated CD8^+^ T lymphocytes, highlighting that HSP70 functions as a DAMP and an effective chaperone for tumor antigens [123].

Hyperthermia significantly augments immunotherapy by facilitating antigen release and diminishing immunosuppressive factors, therefore rendering tumors more susceptible to immune checkpoint inhibitors (ICIs) [129,130,131]. In the ferrimagnetic nanoring MHT model, moderate MHT-induced ICD enhanced tumor sensitivity to PD-L1 inhibition, leading to improved management of both main and distant tumors compared to either treatment in isolation [119]. Extensive reviews indicate that HT can improve ICI pharmacodynamics by augmenting neoantigen burden, facilitating T-cell trafficking, and diminishing suppressive myeloid populations. Although direct bone metastasis models integrating SPION MHT with ICIs have not been explicitly documented, ongoing trials of magnetic hyperthermia in osteosarcoma highlight the potential for effective intrabone thermal therapy [128].

#### 2.5.2. Photothermal Nanoparticles

Near-infrared (NIR)-responsive photothermal nanoparticles transform light into moderate hyperthermia of around 41–45 °C, prompting immunogenic cell death and activating both local and systemic antitumor immunity. Evidence is most robust in solid tumors, particularly in a specific model of lung cancer bone metastasis utilizing a hydrogel system that integrates black phosphorus nanosheets (BPNSs) with a STING agonist [132]. Under NIR irradiation, BPNSs generate hyperthermia and ROS to trigger apoptosis and ICD in tumor cells within osteolytic bone lesions, as demonstrated by surface CRT exposure, nuclear release of high mobility group box 1 (HMGB1), and an approximately five-fold increase in extracellular ATP [132]. These damage-associated molecular patterns (DAMPs) are fundamental characteristics of ICD that stimulate dendritic cell (DC) activation and antigen acquisition [133,134,135,136]. Single-cell RNA sequencing has validated that PTT-induced immunogenic cell death, in conjunction with STING activation, reconfigures the bone metastasis microenvironment, augments immune cell infiltration, and fosters enduring systemic immunity and remote tumor regulation [132].

In nanoparticle-based photothermal therapy (PTT) systems, including gold, carbon, metal, and polymers, mild hyperthermia in the 40–45 °C range promotes apoptosis and necroptosis rather than rapid necrosis. Specifically, temperatures of 43–49 °C trigger apoptotic cell death, while temperatures above 49 °C lead to necrosis [137]. This temperature-regulated stress induces endoplasmic reticulum and mitochondrial stress, resulting in reactive oxygen species generation and enhancing immunogenic cell death signaling in various near-infrared heated nanoplatforms, including gold and other metallic nanoparticles. The effects are amplified when (HSP) responses are limited, as HSPs often provide thermotolerance and mitigate PTT efficacy [137,138,139]. GNR-facilitated targeted hyperthermia at 42–48 °C for five minutes has demonstrated significant ICD in conjunction with chemoradiotherapy and tumor shrinkage in immunologically “cold” malignancies [140].

In the bone metastasis BPNS system, STING agonism enhances tumor cell IFN-β, facilitating the recruitment of immature dendritic cells and augmenting antigen presentation [132]. Upon loading with PTT-generated antigens, dendritic cells activate CD8^+^ cytotoxic T-cells in lymph nodes and augment MHC (Major Histocompatibility Complex Class I) I antigen presentation, particularly when combined with checkpoint blockade or mTOR inhibition [141,142]. ICD-related chemokines, including CXCL9/10 and CCL5, additionally facilitate the recruitment of intratumoral CD8^+^ T-cells [134,143].

In the bone metastasis hydrogel system, photothermal therapy-induced immunogenic cell death combined with STING agonism enhanced immune infiltration in the affected bone and inhibited both local and distant tumors, demonstrating the establishment of memory T-cells [132]. Gold-based and polydopamine-coated platforms exhibit a comparable enhancement of dendritic cell maturation and a reduction in myeloid-derived suppressor cells when photothermal therapy is combined with PD-1/PD-L1 inhibition, effectively “activating” cold tumors [133,140,141,142,144]. Gold nanorods and hybrids demonstrate significant photothermal conversion in the near-infrared spectrum and elicit strong CD8^+^ T-cell responses.

Importantly, the translational maturity of these nanoparticle classes is highly uneven. In oncology more broadly, several nanoplatform categories have already reached regulatory approval or clinical testing, including liposomal formulations, albumin-bound nanoparticles, polymeric micelles, iron oxide nanoparticles for magnetic hyperthermia, and hafnium oxide radioenhancers [145]. However, bone-metastasis-specific nanoparticle strategies remain predominantly preclinical, and direct clinical validation for immune reprogramming within metastatic bone lesions is still lacking [20]. Because the direct clinical validation of nanoparticle-based immunotherapy in bone metastasis remains limited, Table 1 summarizes broader oncology precedents for major nanoparticle platform classes and clarifies how each may inform, but does not yet establish, bone-metastasis-specific nano-immunotherapy. Thus, existing evidence supports the clinical plausibility of multiple nanoplatform classes, but not yet the routine clinical efficacy of bone-targeted nano-immunotherapy as a disease-specific treatment strategy [146]. The translational precedent for major nanoparticle platform classes in oncology and their potential relevance to bone metastasis immunotherapy are summarized in Table 1.

Overall, the major nanoparticle classes discussed in this section differ not only in composition but also in cargo capacity, targeting logic, mechanism of action, and translational readiness. Lipid/liposomal and polymeric systems are the most versatile platforms for loading nucleic acids, cytokines, and combination payloads, and they are particularly attractive when controlled release, co-delivery, or surface functionalization is required; however, they usually need added targeting moieties to achieve reliable skeletal localization in metastatic bone lesions [149]. Bone-targeted ligand-decorated systems, especially those incorporating bisphosphonates or bone-binding peptides, provide the most direct strategy for skeletal accumulation by exploiting hydroxyapatite affinity or lesion-associated binding, although excessive mineral affinity may confine nanoparticles to exposed bone surfaces and limit deeper penetration into tumor-rich marrow compartments [150]. Inorganic photothermal or magnetic platforms add externally controlled functions such as hyperthermia, magnetic guidance, imaging contrast, or spatially triggered activation, making them particularly useful for local immune priming and theranostic applications; nevertheless, their performance depends on lesion accessibility, field or light penetration, and integration with specialized devices [151]. Biomimetic membrane-coated or extracellular-vesicle-inspired systems may improve circulation time, reduce immune clearance, and enhance biological interfacing with tumor or marrow niches, but they remain less standardized with respect to large-scale manufacturing, compositional uniformity, and translational development [152]. Across these categories, translational maturity remains highly uneven: while several nanoplatform classes have already achieved clinical use or clinical testing in oncology, bone-metastasis-specific immunomodulatory nanoparticle systems are still supported mainly by preclinical evidence [153]. Taken together, these platform classes should be viewed as complementary rather than interchangeable, and platform selection should be guided by lesion architecture, intended immune mechanism, and translational feasibility [154]. Representative quantitative outcomes from selected bone-targeted nanoparticle studies discussed across these engineering strategies are summarized in Table 2.

## 3. Immunological Interactions of NPs in the Bone Metastatic Niche

**Figure 3:**
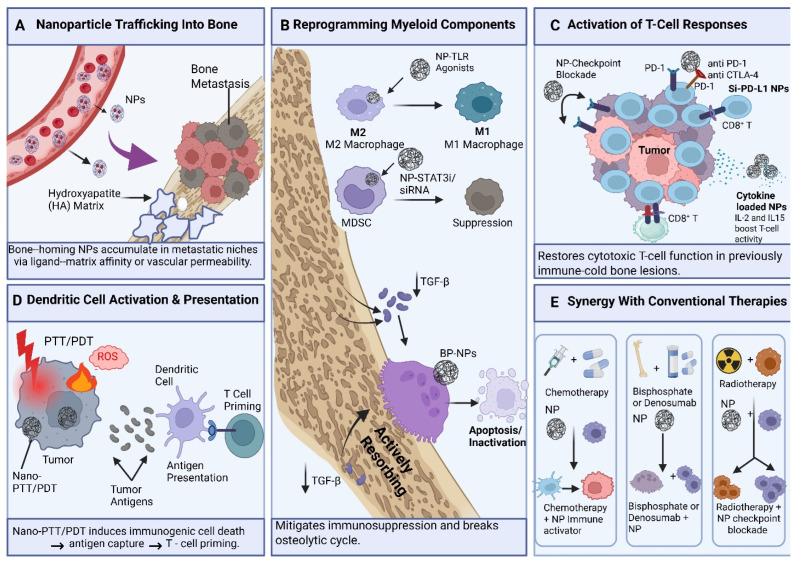
Immunologic mechanisms and therapeutic integration of nanoparticle-based strategies in bone metastases. (**A**) Nanoparticle trafficking into bone: bone-targeting nanoparticles accumulate within metastatic skeletal niches through vascular access and affinity for hydroxyapatite-rich bone surfaces. (**B**) Reprogramming myeloid components: once localized in bone lesions, nanoparticles can repolarize M2-like macrophages toward M1-like phenotypes, suppress myeloid-derived suppressor cells, reduce osteoclast-associated immunosuppression, and help interrupt TGF-β-driven osteolytic immune dysfunction. (**C**) Activation of T-cell responses: nanoparticle platforms can enhance cytotoxic T-cell function through checkpoint blockade, PD-L1 silencing, cytokine delivery, and reversal of T-cell exhaustion within immune-cold bone lesions. (**D**) Dendritic-cell activation and antigen presentation: photothermal or photodynamic therapy and adjuvant-loaded systems promote immunogenic cell death, antigen release, dendritic cell maturation, and downstream T-cell priming. (**E**) Synergy with conventional therapies: these immune effects can be integrated with chemotherapy, bisphosphonates or denosumab, and radiotherapy to simultaneously reduce tumor burden, preserve bone structure, and improve antitumor immunity. Overall, the figure illustrates how nanoparticle-mediated trafficking, immune remodeling, and combination treatment can cooperatively disrupt the tumor–bone vicious cycle. Created in BioRender. Mohammad, K. (2026) https://BioRender.com/gd9qpxo (accessed on 22 April 2026).

The major immune mechanisms through which bone-targeting nanoparticles traffic to skeletal lesions, remodel myeloid suppression, promote dendritic-cell priming, restore T-cell activity, and synergize with standard therapies are summarized in Figure 3.

### 3.1. Myeloid Cell Modulation: Reprogramming TAMs from M2 to M1

Nanoparticle administration of toll-like receptor (TLR) agonists can effectively convert tumor-associated macrophages (TAMs) from M2-like to tumoricidal M1 phenotypes and restructure immunosuppressive microenvironments. Although direct data in the bone metastatic niche are limited, evidence from several solid tumor models indicates that these processes are highly transferable. For example, platforms administering R848 (TLR7/8 agonist) or R837 (imiquimod) exhibit significant M2 to M1 repolarization of tumor-associated macrophages (TAMs). In the absence of T and NK cells, β-cyclodextrin nanoparticles loaded with R848 (CDNP-R848) have demonstrated the ability to induce tumor regression via the re-education of pro-inflammatory tumor-associated macrophages (TAM) [159]. Transcriptional reprogramming through TLR signaling predominantly entails the NF-κB, signal transducer and activator of transcription 1 (STAT1), and interferon regulatory factor (IRF) pathways. Endosomal TLR7/8 agonists enlist myeloid differentiation primary response 88 (MyD88) to activate NF-κB and activator protein 1 (AP-1), resulting in elevated production of IL-6, interleukin-12 (IL-12), and TNF-α [160]. TLR3 agonists, including polyinosinic:polycytidylic acid [poly(I:C)], activate TIR-domain-containing adapter-inducing interferon-β (TRIF) to signal TBK1 and IRF3, resulting in the induction of type I interferon and costimulation in macrophages and dendritic cells, hence augmenting CD8^+^ T-cell priming. The amalgamation of poly(I:C) and R848 within nanocapsules exhibits enhanced efficacy by connecting TLR–JAK/STAT1–IRF pathways to sustained M1 reprogramming [161]. This myeloid re-education counteracts immunosuppression by altering cytokine and chemokine profiles; M1-polarized (TAMs) and activated (DCs) release IL-12 and type I interferons, which recruit CD8^+^ T-cells while reducing IL-10 and ARG1 levels [159,160,162,163,164,165]. Moreover, TLR7/8 agonists diminish MDSC accumulation by transforming them into macrophages or dendritic cells, thus mitigating local suppression [160,164].

The reprogrammed myeloid cells additionally augment dendritic cell development and antigen presentation. NP-delivered R848 or 3M-052 significantly enhances costimulatory receptors, including CD80 and CD86, hence augmenting CD8^+^ T-cell priming in both lymph nodes and tumors [160,166,167]. This alteration in the immunological environment promotes enhanced CD8^+^ T-cell infiltration and diminished Treg populations, yielding sustained systemic immunity and metastasis regulation [162,164,165,166,167,168]. A TLR agonist MOF NP modified with a zoledronic acid bisphosphonate-modified Toll-like receptor (TLR) agonist-integrated nanoscale metal–organic framework has exhibited significant upregulation of macrophage CD86 and effective prevention of bone metastases in mice, indicating the potential applicability of these NP-driven techniques to skeletal lesions [169].

### 3.2. Enhancing T-Cell Recruitment and Function

#### 3.2.1. Local Delivery of Immune-Stimulatory Cytokines

While not exclusive to bone, numerous studies illustrate how localized cytokine reservoirs modify immune responses. Intratumoral lipid nanoparticles (LNPs) that encode IL-12 and interleukin-27 (IL-27) elicit significant local cytokine production and substantial infiltration of interferon-γ (IFN-γ)/TNF-α^+^ CD8^+^ T-cells and NK cells, all while avoiding systemic damage [170]. IL-12 induces Th1/Tc1 responses through STAT4, but IL-27 enhances effector cell survival. Collagen-binding IL-12 (CBD-IL-12) and CBD-granulocyte-macrophage colony-stimulating factor (GM-CSF), designed to adhere to tumor stroma, exhibit extended retention in the microenvironment, reprogram tumor-associated macrophages, and synergistically enhance the proliferation of tumor-resident CD8^+^ T-cells [171]. Tumor-targeted interleukin-2 (IL-2) derivatives and immunocytokines increase IL-2 concentration in the tumor microenvironment, augmenting effector activation while restricting regulatory T-cells and systemic toxicities [172,173]. These matrix- or stroma-anchored cytokines function as reservoirs that maintain elevated local concentrations to preferentially activate tumor-infiltrating immune cells [170,171,172,174].

Limiting cytokine biodistribution is essential, as systemic therapy like high-dose IL-2 or IL-12 is associated with cytokine release syndrome (CRS) and vascular leakage, resulting in multiorgan damage [173,175,176,177]. By limiting cytokine dosage and localization, nanoparticles can diminish off-target exposure and decrease the likelihood of cytokine release syndrome associated with free cytokines [178]. Analogous to collagen-binding systems, a BP-functionalized or bone-collagen-targeted nanoparticle delivering IL-12, IL-2, or GM-CSF to marrow metastases is anticipated to elevate local cytokine concentrations within the niche (trabecular bone, marrow stroma, endosteal surfaces) while maintaining low systemic levels. This would enhance the local activation of CD8^+^ T-cells and NK cells present in or migrating through the marrow, similar to the heightened effector infiltration observed with localized mRNA LNPs [170,171].

Moreover, this strategy could augment dendritic cell activation and cross-priming in the bone marrow and draining lymph nodes. This is especially pertinent when GM-CSF and IL-12 are co-administered, as CBD-GM-CSF has demonstrated the ability to upregulate IL-12 receptors and STAT4 signaling in dendritic cells [171]. Nonetheless, data gaps persist; no existing study has yet shown BP or bone collagen-targeted nanoparticles delivering these specific cytokines to bone metastases, while current bone-targeted nanoparticles predominantly concentrate on chemotherapeutics or kinase inhibitors [17,27,35,40]. The function of GM-CSF in bone is intricate, as it can either stimulate antitumor myeloid activation or enhance MDSCs, contingent upon dosage and context, requiring precise modulation of local concentrations [170,179].

#### 3.2.2. Reversal of T-Cell Exhaustion

Evidence for RNA delivery targeting bone originates from three areas: the manufacturing of bone-specific nanocarriers, the nanoparticle delivery of checkpoint-axis payloads, and mechanistic insights into the ways genetic regulation alters the metabolism and epigenetics of fatigued CD8^+^ T-cells. No comprehensive study has yet combined all these elements to reprogram tired T-cells in bone metastases; nonetheless, the individual technological components are well-established.

Polymeric and lipid systems can be designed for accumulation in bone and marrow. ALN-Pabol, a bioreducible polymer containing alendronate, binds hydroxyapatite with 91% effectiveness, surpassing traditional lipid nanoparticles. In models of breast cancer bone metastases, ALN-Pabol/miRNA formulations decreased tumor weight by 79.1% and reinstated bone architecture [158]. Likewise, branched polyethylenimine (PEI)–alendronate conjugates (PCA/miR-34a) have exhibited improved bone accumulation and inhibition of osteolysis [180]. In addition to bisphosphonate-modified systems, lipid–polymer nanoparticles tailored for bone marrow endothelium can effectively transport siRNA to inhibit cyclophilin A (CyPA), hence diminishing tumor burden in vivo [181]. These technologies validate that nucleic acid payloads can be directly administered into the metastatic niche to target tumor, stromal, and immunological populations [158,180,181,182].

The engineering viability of PD-L1 or T-cell-targeted RNA delivery is substantiated by platforms like bispecific antibody-anchored lipid nanoparticles. These constructions utilize antibodies that recognize targets such as PD-L1, CD4, or CD5 to facilitate target-specific mRNA transfection, with T-cell-directed forms increasing transfection efficiency by up to 26-fold [183]. Alternative techniques include lipid nanoparticles (LNPs) coated with membranes derived from PD-1-overexpressing cells to specifically target PD-L1^+^ tumors while concurrently disrupting the inhibitory axis [184]. Moreover, clustered regularly interspaced short palindromic repeats/CRISPR-associated protein 9 (CRISPR/Cas9) lipid nanoparticles have effectively been employed to modify genes in hematopoietic and leukemic cells within the bone marrow [182]. The observations indicate that bone-targeting LNPs may be further modified with PD-L1 or T-cell-directed ligands to specifically target genetic editing within bone metastases.

Multiple systems currently provide miRNAs to skeletal malignancies to reinstate tumor-suppressive pathways. CaP nanoparticles administering miR-205-5p to bone metastatic prostate cancer cells suppress oncogenic targets, including AR and ZEB1, resulting in tumor cell apoptosis [185]. Although these investigations concentrate on tumor-intrinsic pathways, they exhibit effective delivery into bone-resident cells, indicating that these platforms may be repurposed to deliver miRNAs that influence T-cell fatigue or metabolic regulators in situ [158,180,185,186].

Mechanistic studies on T-cell fatigue indicate that genetic modification may alter CD8^+^ functionality by targeting the persistent transcriptional and epigenetic reprogramming that enforces malfunction [187,188,189,190,191]. Exhaustion is associated with non-coding RNA (ncRNA)-mediated regulation of chromatin enzymes and metabolic reconfiguration, wherein metabolites like lactate and acetyl-CoA function as cofactors for histone and DNA-modifying enzymes [188,191,192]. Persistent PD-1/PD-L1 signaling induces mitochondrial dysfunction that contributes to this condition [187,188,189].

Thus, a bone-targeted nanoparticle delivery system could be implemented to deliver a “payload stack” of siRNA/CRISPR targeting PD-L1 or T-cell immunoglobulin and mucin-domain containing-3 (TIM-3) to diminish inhibitory signaling and improve mitochondrial function, in conjunction with miRNAs that target transcription factors or metabolic pathways associated with exhaustion [188,191,192,193]. This multifaceted strategy may reactivate chromatin, reinstate cytotoxic gene programs, and enhance oxidative capability within the bone metastatic niche [185,188,192,194]. Current research mostly concentrates on tumor cells and osteoclasts; however, the literature offers a cohesive framework for employing these genetic tools to enhance sustained effector activity within bone-resident immune compartments [158,180,181,185,186].

### 3.3. Activating Dendritic Cells (DCs)

#### 3.3.1. Nanovaccines

Current data demonstrate the feasibility that bone-targeted nanovaccines, co-delivering tumor antigens with STING agonists or CpG, could leverage conventional type 1 dendritic cells (cDC1s)-mediated cross-priming and BMDC cross-dressing to elicit systemic CD8^+^ anti-tumor immunity; however, this requires direct testing in bone-focused models.

Evidence robustly indicates that nanovaccines co-delivering tumor antigens alongside STING agonists or cytosine-phosphate-guanine (CpG) oligonucleotide serve as potent inducers of antigen cross-presentation and CD8^+^ T-cell priming. Although direct data on bone-targeted variants operating particularly within the bone marrow niche are currently lacking, mechanistic insights can be inferred from the biology of BMDC and conventional dendritic cells type 1 (cDC1). Cross-priming transpires when cDC1s direct foreign antigens into the MHC I pathway, whereas cross-dressing entails dendritic cells acquiring and presenting pre-formed peptide–MHC I complexes from other cells, including apoptotic tumor cells [195,196,197,198]. These processes are pivotal to effective immunotherapy and rely on costimulation and cytokine assistance [195,199].

Multiple STING agonist nanovaccines co-encapsulating peptide antigens exhibit selective uptake by cDC1s, subsequently leading to endosomal escape and enhanced surface presentation of MHC I–antigen complexes. These platforms stimulate substantial production of type I IFN, TNF-α, and CXCL9/10, facilitating the proliferation of antigen-specific CD8^+^ effector and memory T-cells [200,201]. A cDC1-targeted silica nanovaccine (Si9GM) employing BMDC membranes and 2′3′-cGAMP specifically targets C-type lectin domain family 9 member A (CLEC9A) cDC1s to enhance cross-presentation [200]. Likewise, pH-responsive vesicular nanoparticles that co-deliver cGAMP and monophosphoryl lipid A (MPLA) synergistically augment DC costimulatory indicators and peptide cross-presentation, resulting in enhanced tumor control [202].

CpG-based nanovaccines, which are TLR9 agonists, have a comparable benefit. When encapsulated in nanocarriers containing tumor cell-membrane antigens, CpG enhances endocytosis and cross-presentation in BMDCs, leading to maturation and strong anti-tumor responses when paired with anti-PD-L1 [203]. Photothermal nanovaccines employing CpG and IDO inhibition have demonstrated the capacity to generate in situ antigen release and systemic CD8^+^ responses, hence facilitating long-term memory against metastatic lesions [204]. The results indicate that associating tumor antigens with dendritic cell maturation through TLR9 is highly effective, despite the less frequent examination of specific MHC I processing steps compared to STING models [203,204,205].

In the bone marrow niche, these processes are anticipated to function through multiple coordinated stages. Initially, bone-targeting nanovaccines would be absorbed by marrow-resident dendritic cell progenitors or cDC1-like cells, employing pH-responsive or PEI-containing carriers to facilitate endosomal escape into the cytosol for proteasomal processing [200,201,202,206]. Secondly, the intrinsic signaling from STING or TLR9 functions as a “licensing” signal, transforming immature antigen-presenting cells into proficient cross-priming cells by enhancing MHC and costimulatory molecule expression [201,203,207]. Third, this inflammatory milieu may augment cross-dressing mechanisms, wherein dendritic cells obtain complexes from tumor cells that have experienced immunogenic death [196,197,200].

This local marrow priming would ultimately result in an increase in systemic CD8^+^ T-cell repertoires. Activated cDC1s may move to draining lymph nodes or directly engage with recirculating naïve T-cells in the marrow and spleen, whereas STING-induced chemokines such as CXCL9/10 attract these effectors back into bone lesions [195,199,201]. Although this model has yet to be explicitly evaluated in bone-centric investigations, the alignment of BMDC and cDC1 data positions the development of bone-targeted nanovaccines as a critical research goal.

#### 3.3.2. In Situ Vaccination at Bone Lesions

In situ vaccination aims to convert the metastatic bone lesion into a personalized vaccine reservoir. Local PTT/Photodynamic therapy (PDT).

elicits ICD, resulting in the release of a diverse array of tumor-specific antigens, neoantigens, and danger signals, while antigen-capturing NPs collect and transmit these antigens to dendritic cells (DCs) for systemic T-cell priming [133,208,209,210,211,212]. PTT/PDT produces endoplasmic reticulum stress, reactive oxygen species production, and hyperthermia, resulting in calreticulin exposure, ATP and HMGB1 release, and cell death through ferroptosis or apoptosis. In bone metastasis, agents such as black phosphorus nanosheets or gold-based systems have effectively ICD to transform the “cold” bone microenvironment and enhance the efficacy of STING agonists or checkpoint inhibitors [132,133,210].

Antigen-capturing nanoparticles are designed to bind and safeguard endogenous neoantigens generated during immunogenic cell death, addressing the challenges of dilution and fast proteolysis [208,209,212,213]. Various capture chemistries are presently employed:

The maleimide–thiol Michael addition involves FeO_x_ nanoadjuvants or liposomes (L-Mals) with surface maleimide that effectively “click” with free sulfhydryl groups on tumor antigens generated by radiation or photothermal therapy, hence optimizing autoantigen bioavailability [209,213].

PEI-based nanoparticles employ positive charges and pyridyl disulfide groups for disulfide exchange with antigens. Furthermore, PBA “nanochaperones” sequester glycoprotein neoantigens liberated by PDT via reversible covalent and hydrophobic contacts, thereby protecting them from proteolytic enzymes [212].

The objective of the design is to enhance local antigen density on an immunogenic surface while simultaneously co-delivering adjuvants (CpG, R848, STING agonists) to promote dendritic cell uptake and lymph node trafficking [208,209,212,213,214].

PTT and PDT produce polyclonal neoantigen pools derived from mutant proteins, aberrant glycoproteins, and oxidatively changed self-proteins [133,210,211,212,215]. Soluble fragments, membrane debris, and exosomes are released within the bone lesion. Antigen-capturing nanoparticles administered intratumorally encounter abundant exposed cysteines, lysines, and glycan motifs on denatured proteins. By creating NP–neoantigen complexes, they aggregate several antigens on a nanoscale scaffold, enhancing affinity for dendritic cell receptors and directing antigen trafficking into MHC I cross-presentation pathways [208,209,212,213,216].

Immunogenic cell death in bone metastases releases ATP as a “find-me” signal alongside DAMPs such as CRT and high mobility group box 1 (HMGB1), which attract and activate dendritic cells in the bone marrow and perilesional bone [132,133,210,211,215]. Antigen-loaded NPs provide pathogen-mimicking signals—such as CpG or STING agonists—that activate TLR 7/8, TLR9, or the cGAS–STING pathway in DCs. This process enhances DC maturation, characterized by upregulation of CD80/86 and MHC II, and increases interleukin-12 (IL-12) production [132,209,214,216,217,218]. These mature DCs subsequently migrate to draining lymph nodes, transporting captured neoantigens to prime cytotoxic CD8^+^ T-cells and T helper 1 (Th1) cells, which then return to both the bone lesion and distant metastases [132,133,208,209,210,213,214]. In models of bone metastasis, this combination of thermal/oxidative ICD and antigen-capturing NPs has been shown to enhance DC activation, increase CD8^+^ T-cell infiltration, and generate long-term immune memory while concurrently facilitating bone repair [132,133,210].

## 4. Gene and RNA Delivery

### 4.1. Nucleic Acid-Based Nanotherapeutics for Modulating Bone Metastatic Pathways

The transcriptional, immunoregulatory, and tumor–bone signaling pathways that control metastatic competence and immune evasion can be directly targeted with gene- and RNA-based nanotherapeutics. Many of these pathways remain out of reach for conventional small-molecule drugs. In bone metastases, however, systemic delivery of nucleic acids is particularly difficult because they are quickly degraded by nucleases, taken up inefficiently by cells, and have poor access to lesions within the marrow niche [157]. Nanoparticle carriers help overcome these barriers by shielding nucleic acids from degradation, allowing control over particle size and surface charge to improve circulation, and incorporating bone-targeting ligands that promote accumulation at mineralized metastatic sites [219] (Figure 1C).

#### 4.1.1. siRNA Delivery Targeting Metastatic and Osteo-Modulatory Pathways

siRNA-based nanotherapeutics have been used to suppress transcription factors and signaling pathways that drive epithelial-to-mesenchymal transition (EMT), invasion, and metastatic persistence, thereby lowering tumor cell fitness for bone colonization [157]. engineered PEGylated chitosan nanoparticles for the delivery of twist1 siRNA, comparing a non-targeted formulation with an alendronate-functionalized variant designed to exploit hydroxyapatite affinity for bone targeting [157]. Both formulations showed hydrodynamic diameters below 70 nm and near-neutral ζ-potentials—physicochemical properties that support systemic administration and reduce nonspecific protein interactions [157].

Encapsulating siRNA within nanoparticles substantially improved its stability in serum, with intact siRNA detectable for up to 6 h in PEGylated chitosan nanoparticles, compared with the rapid degradation of naked siRNA [157]. Nanoparticle-mediated delivery resulted in significant TWIST1 protein knockdown in 4T1 breast cancer cells and produced a functional phenotype consistent with reduced metastatic potential, as evidenced by impaired directional migration in wound-healing assays. Together, these findings show that siRNA-loaded nanoparticles can drive both molecular and phenotypic suppression of EMT-associated programs relevant to metastatic spread [157].

Beyond tumor-intrinsic pathways, siRNA nanodelivery has also been used to disrupt reciprocal tumor–bone signaling. Sonic Hedgehog (SHH) signaling promotes prostate cancer bone metastasis by stimulating osteoclast differentiation and aberrant osteoblast activity [219]. Liu et al. developed a multifunctional nanoplatform consisting of a ZIF-8 core loaded with SHH siRNA, a lipid shell containing docetaxel, and alendronate-functionalized surface ligands for bone targeting [219]. This dual-delivery system inhibited prostate cancer cell proliferation, induced G2/M arrest and apoptosis in vitro, and preferentially accumulated at bone metastatic sites in vivo, where it significantly reduced tumor growth with minimal systemic toxicity [219]. Together, these findings support a dual-action paradigm in which siRNA-mediated disruption of osteo-modulatory signaling is combined with cytotoxic therapy to suppress bone metastatic progression [219].

#### 4.1.2. mRNA Nanoparticles for Immune Activation in Bone Metastasis

mRNA nanotherapeutics offer a transient yet potent strategy for inducing the local expression of immunostimulatory proteins, an approach that is particularly attractive in bone metastases, where sustained systemic cytokine exposure is associated with dose-limiting toxicity [220]. Ma et al. developed an mRNA tumor vaccine incorporating MPLA as an intrinsic Toll-like receptor 4 agonist to enhance dendritic cell activation and antigen presentation [220]. Administration of the mPLA/mRNA formulation induced dendritic cell maturation, macrophage polarization toward an M1 phenotype, and increased secretion of IFN-γ and IL-12, cytokines associated with effective antitumor immunity [220].

Notably, immune activation elicited by the mRNA nanovaccine translated into reduced metastatic burden in bone lesions, in addition to suppression of primary tumor growth, demonstrating that systemically induced immune responses can exert meaningful control over skeletal metastases [220]. These results support the feasibility of mRNA nanomedicine as a strategy to transiently reprogram immune tone in a way that counteracts bone marrow-associated immunosuppression [220].

#### 4.1.3. CRISPR/Cas9 Nanoparticle Systems for Durable Pathway Reprogramming

Nanoparticle-mediated delivery of CRISPR/Cas9 components enables a permanent disruption of the genes that drive immune evasion and metastatic progression, without the need for viral vectors [221,222]. Mao et al. developed lipid-assisted nanoparticles for the co-delivery of Cas9 mRNA and PD-L1 single-guide RNA (sgRNA) to dendritic cells, achieving efficient intracellular delivery and genome editing, with PD-L1 mutation frequencies reaching 16.6% as determined by deep sequencing [221]. The edited dendritic cells exhibited enhanced T-cell stimulatory capacity, which in turn led to suppressed tumor growth in vivo [221]. Although this approach has not yet been evaluated in bone metastasis models, it provides a mechanistic blueprint for reprogramming antigen-presentation pathways that are suppressed within the bone metastatic niche [221].

Complementing these immune-focused genome-editing strategies, Fieni et al. reported a lipid nanoparticle system delivering CRISPR components targeting interleukin-30 (IL-30), a cytokine implicated in prostate cancer metastasis and tumor–stromal interactions [222]. Systemic administration of this platform significantly reduced metastatic dissemination in vivo and was accompanied by downregulation of pro-metastatic mediators, including CXCR4, a key regulator of bone homing [222]. Together, these studies demonstrate that nanoparticle-enabled CRISPR delivery can durably reprogram immune and metastatic pathways, supporting its future application in bone metastasis, where sustained therapeutic effects are likely to be required [221,222].

### 4.2. Photothermal (PTT) and Photodynamic Therapy (PDT)

#### 4.2.1. Mechanistic Basis: Nanoparticle-Enabled ICD and Antigen Release

Nanoparticle-mediated PTT relies on photothermal agents, such as gold nanorods or polydopamine, that efficiently convert near-infrared light into localized heat, inducing tumor cell death while minimizing damage to surrounding bone marrow when properly controlled [223]. Gu et al. developed a photothermal–chemotherapeutic platform that combines gold nanorods (GNRs) with thermosensitive liposomes, enabling on-demand drug release under mild hyperthermia [223]. In vitro, near-infrared-triggered PTT induced tumor cell apoptosis and necrosis, whereas in vivo application in a bone metastasis model produced effective tumor ablation while preserving bone structure, demonstrating spatially confined cytotoxicity that is compatible with skeletal tissues [223].

Photodynamic therapy similarly induces ICD through the generation of ROS following light activation of photosensitizers, leading to oxidative damage of tumor cell membranes and organelles [224]. Liu et al. reported a transformable supramolecular nanoparticle carrying the photosensitizer chlorin e6, which generated robust ROS upon laser irradiation and induced classical ICD markers, including increased CRT exposure on tumor cells and extracellular HMGB1 release [224]. Quantitative immunofluorescence and flow cytometry analyses showed increased CRT and HMGB1 levels in treated tumors compared with non-irradiated controls, directly linking nanoparticle-mediated PDT to the induction of ICD [224].

Importantly, ICD triggered by nano-PTT or nano-PDT resulted in enhanced antigen availability and DC activation. In the PDT study by Liu et al., treatment significantly increased intratumoral populations of CD11c^+^CD80^+^ and CD11c^+^CD86^+^ DCs, consistent with enhanced antigen uptake and maturation following ICD [224]. These mechanistic data support the view that nanoparticle-enabled phototherapies can act as in situ vaccination strategies, a concept that is particularly relevant for bone metastases, where endogenous antigen presentation is typically weak [224,225].

#### 4.2.2. Combined Nano-PTT and Immunotherapy

Although nano-PTT and nano-PDT induce ICD, immune activation is frequently dampened by checkpoint-mediated T-cell exhaustion and immunosuppressive pathways such as indoleamine 2,3-dioxygenase 1 (IDO-1) and TGF-β, which are abundant in bone metastatic niches [224,226]. Consequently, recent primary studies have increasingly focused on combining phototherapies with immune modulators, delivered either systemically or within a single nanoparticle platform.

A representative example is the work by Liu et al., who developed a transformable supramolecular nanoparticle (NLG919@CF) that integrates PDT with immune modulation by encapsulating NLG919, an IDO-1 inhibitor, and combining treatment with systemic anti-PD-1 antibody [224]. Upon laser irradiation, the nanoparticle generated ROS, induced ICD, and transformed from spherical nanoparticles into nanofibers, increasing intratumoral retention [224]. In vivo, the triple-combination therapy (NLG919@CF + laser + anti-PD-1) reduced tumor growth to 25.1% relative tumor volume, compared with 100% in PBS-treated controls, significantly outperforming PDT or checkpoint blockade alone [224].

Immunologically, this combination markedly increased cytotoxic T lymphocyte (CTL) infiltration, with a reported 19.21-fold increase in CD8^+^ T-cells relative to controls, and improved the CTL-to-regulatory T-cell ratio within the tumor microenvironment [224]. Importantly for skeletal disease, micro-computed tomography analysis demonstrated significantly reduced osteolysis in bone metastatic lesions, with tibial bone volume increased 1.26-fold relative to untreated controls, and tartrate-resistant acid phosphatase staining confirmed reduced osteoclast activity [224]. These data provide direct evidence that nano-PDT combined with checkpoint blockade can concurrently enhance antitumor immunity and preserve bone integrity [224].

Complementary strategies have investigated photothermal nanoparticles as carriers for cytokine delivery to enhance immune priming. Polydopamine nanoparticles, in particular, have emerged as attractive photothermal agents owing to their biocompatibility and high photothermal conversion efficiency [226]. In primary tumor models with metastatic relevance, polydopamine-based photothermal nanoparticles loaded with interleukin-12 (IL-12) have been shown, upon irradiation, to induce robust immunogenic cell death while locally releasing IL-12, leading to enhanced dendritic cell activation, increased IFN-γ production, and expansion of tumor-infiltrating CD8^+^ T-cells [226]. Although these studies were not conducted exclusively in bone metastasis models, the mechanistic synergy between photothermal ablation and localized cytokine delivery directly addresses key immunosuppressive features of the bone metastatic microenvironment [226].

### 4.3. Nanoparticle-Mediated Checkpoint Blockade

Immune checkpoint inhibitors targeting the PD-1/PD-L1 axis have transformed cancer therapy; however, clinical and translational evidence indicate that bone metastases frequently respond less effectively than many visceral lesions and are associated with worse outcomes in ICI-treated cohorts [227,228,229]. 

Real-world organ-level analyses in metastatic non-small cell lung cancer (NSCLC) further suggest that bone lesions can respond discordantly compared with visceral disease under PD-1 blockade and that the presence of bone metastases is an independent adverse prognostic factor for overall survival [227].

Together, these observations position skeletal disease as a site where “standard” checkpoint pharmacology is often insufficient unless the bone-specific resistance programs are concurrently neutralized [227,228,229].

#### 4.3.1. Organ-Specific and Systemic Immunologic Consequences of Bone Metastasis

Beyond local under-response at skeletal sites, emerging evidence indicates that bone metastases can systemically suppress antitumor immunity and diminish checkpoint responsiveness at extraosseous tumor sites [230]. In a 2025 mechanistic study, bone metastases were shown to condition osteoclasts to produce osteopontin, a circulating mediator that impairs antitumor T-cell states and drives resistance to immune checkpoint blockade in extraosseous tumors. Targeting osteoclasts or osteopontin restored sensitivity to checkpoint therapy [230]. This “bone-to-systemic” axis implies that effective checkpoint strategies for bone-metastatic patients may require not only local activation in bone lesions but also interruption of bone-derived immunosuppressive signals with endocrine-like reach [230].

#### 4.3.2. Bone Metastatic Niche Mechanisms That Blunt PD-1 and PD-L1 Efficacy

The bone marrow metastatic niche is enriched in myeloid lineages and fosters immune states that restrict effective cytotoxic T-cell infiltration and function, including expansion of tumor-associated macrophages and/or MDSCs and the induction of T-cell dysfunction [229]. 

Single-cell profiling of human prostate cancer bone metastases revealed pronounced shifts toward tumor-associated inflammatory monocytes and macrophages at tumor sites, accompanied by diminished cytotoxicity signatures and a strong exhaustion phenotype in a tumor-proximal CTL subset, with myeloid abundance correlating with CTL dysfunction [231].

In syngeneic 4T1 breast cancer bone metastasis, bone lesions exhibited increased functional polymorphonuclear and monocytic MDSCs, and monocytic MDSCs were predominantly PD-L1^+^ within bone metastases, consistent with local suppression of PD-1^+^ T-cells [232].

Mechanistically, suppressed (non-activated) tumor-infiltrating T-cells can become pro-osteoclastogenic—via increased TNF-α and RANKL—thereby amplifying osteoclast formation and osteolysis, whereas activated T-cells can exert anti-osteoclast effects through cytokines such as IFN-γ and IL-4 [232].

Taken together, these data suggest that PD-1/PD-L1 blockade in bone metastasis is limited not only by checkpoint signaling itself but also by the myeloid-suppressive and bone-remodeling circuits that reinforce coupled T-cell suppression and osteoclast activation [232].

TGF-β represents an additional resistance hub with direct relevance to bone metastases because osteoclastic resorption can enrich local TGF-β activity, reinforcing immune exclusion and suppression [220,224].

In immune-excluded tumors, TGF-β signaling has been shown to attenuate response to PD-L1 blockade by restricting T-cell penetration into the tumor compartment, while co-inhibition of TGF-β and PD-L1 reduces stromal TGF-β signaling and promotes T-cell infiltration with improved tumor control [224].

Therefore, checkpoint strategies for skeletal disease may require deliberate disruption of TGF-β-driven exclusion phenotypes that are plausibly amplified in the resorptive bone metastatic niche [220,224].

Finally, osteoclast-lineage cells can directly impose checkpoint-like suppression that is not rescued by PD-1 blockade alone [233].

Osteoclast-derived apoptotic bodies have been shown to suppress naïve CD8^+^ T-cell activation through sialic acid-binding immunoglobulin-like lectin 15 (Siglec-15)-dependent inhibition of co-stimulatory signaling, and Siglec-15 neutralization reduced secondary metastases and improved survival in a breast cancer bone metastasis model [233].

These findings suggest that multi-layered checkpoint networks—spanning both adaptive and osteoclast/myeloid checkpoints—may need to be co-targeted to fully restore cytotoxic T-cell function in bone-metastatic disease [233]. In bone metastasis, PD-L1 is expressed not only by tumor cells but also by suppressive myeloid cells, where it helps inhibit T-cell function. As a result, PD-1/PD-L1 blockade may counter both tumor-driven and myeloid-mediated immune suppression. However, systemic or nonselective blockade can cause off-target immune effects and may still be insufficient if other suppressive pathways, such as TGF-β, osteoclast-related dysfunction, or Siglec-15, remain active. Bone-directed nanocarriers are therefore attractive because they can localize checkpoint inhibition and help reprogram the immunosuppressive niche.

#### 4.3.3. Nanoparticle-Enabled Strategies That Mechanistically Sensitize Bone Lesions to Checkpoint Blockade

Current nanoparticle designs represent a shift from passive delivery to active immune engineering. By integrating ICD-inducing agents to drive antigen release [234], metabolic modulators to reverse myeloid-driven [235], and gene-silencing payloads to short-circuit adaptive resistance loops [234,236], these platforms provide a multi-modal solution to the immune-excluded landscape of bone metastases.

In a breast cancer bone metastasis model, a transformable supramolecular nanoparticle that combined a photosensitizer for ROS-mediated tumor killing with an IDO-1 inhibitor to alleviate Treg-mediated suppression enhanced cytotoxic T-cell function and synergized with anti-PD-1 antibody therapy. These findings support the concept that effective checkpoint blockade in bone metastasis requires coordinated local immune priming together with relief of intralesional immunosuppression [224].

In prostate cancer bone metastases, biomimetic stem-cell-membrane-coated nanoparticles co-delivering doxorubicin and PD-L1 siRNA exemplify a complementary mechanism: chemotherapy-enhanced antigen release coupled with local PD-L1 knockdown to prevent adaptive PD-L1 upregulation and restore T-cell activity within the bone niche [234].

More broadly, tumor-targeted nanomedicine co-delivering anti-PD-1 with gene-silencing payloads that reprogram TAM/MDSC immunometabolism demonstrates how nanoparticle designs can coordinate checkpoint inhibition with active reversal of myeloid-driven immunosuppression, a feature expected to be particularly relevant in myeloid-rich bone metastases [235].

Given the central role of TGF-β-driven immune exclusion as a resistance mechanism, nanovesicles that co-deliver a TGF-β receptor inhibitor together with photodynamic immune priming and additional suppression of inducible PD-L1 offer a mechanistic template for integrating “de-exclusion + priming + checkpoint suppression” modules. This framework could be adapted to TGF-β-enriched skeletal metastases [236].

#### 4.3.4. Spatial Confinement, Bone Targeting, and Therapeutic-Index Logic

Systemic PD-1/PD-L1 blockade can cause immune-related adverse events that complicate sustained dose intensification and constrain therapeutic flexibility, underscoring the need for strategies that enhance local efficacy without proportionally increasing systemic exposure [237]. 

Bone-targeting delivery principles are supported by antibody-engineering studies in which adding bone-targeting peptide sequences significantly enriched antibody accumulation in bone tumor sites and was proposed to enhance local antitumor activity while decreasing systemic toxicity, providing a clear translational rationale for bone-targeted checkpoint formats [238].

Locoregional and bone-adjacent biomaterial strategies further support safety-oriented checkpoint design in skeletal disease; for example, a thermosensitive injectable hydrogel combining biomimetic mineral cues with anti-CD47 promoted M1 macrophage polarization, activated CD8^+^ T-cells, suppressed tumor growth, and reduced bone resorption in a prostate cancer bone metastasis model while aiming to avoid severe side effects associated with conventional macrophage activators [239].

### 4.4. Nanoparticle-Enabled Cancer Vaccination Strategies

Although these vaccination strategies have not yet been tested specifically in bone metastasis models, their ability to generate systemic cytotoxic T-cell immunity suggests clear potential for application to skeletal metastatic disease, where endogenous antigen presentation is often limited. Nanovaccine platforms are designed to improve the spatial and cellular coordination of cancer vaccination by co-delivering antigen and immune adjuvants to professional antigen-presenting cells, particularly lymph node-resident and migratory dendritic cell subsets optimized for cross-presentation to CD8^+^ T-cells [206]. In a 2024 Bioactive Materials study, Nguyen et al. engineered a dendritic cell membrane-coated silica nanovaccine (Si9GM) that targets cDC1 via anti-CLEC9A while co-delivering antigen (OVA_7–264) and the STING agonist 2′3′-cGAMP. This platform enhanced DC maturation, boosted antigen-specific CD8^+^ T-cell responses, reduced intratumoral Tregs, and produced antitumor effects that were further improved when combined with anti-PD-1 therapy [200].

A complementary “in situ vaccination” strategy uses nanomaterials to convert the tumor itself into an endogenous antigen source while simultaneously supplying the innate immune activation needed for effective T-cell priming [240]. In 2024, Li et al. reported a hybrid nanoparticle in situ vaccine that coupled intratumoral immune activation with the protection of dendritic cells from oxidative dysfunction by integrating ROS scavenging with STING pathway activation. This design strengthened downstream T-cell activation and systemic antitumor immunity in preclinical models [240].

Beyond peptide-based and in situ approaches, mRNA nanovaccines can trigger intratumoral T-cell inflammation and establish immunologic memory, even in tumors that are initially immunologically “cold” [241,242]. In 2025, Fournier et al. demonstrated that an mRNA vaccine formulated in a nanostructured lipid carrier remodeled the tumor microenvironment toward a T-cell-inflamed phenotype and supported durable antitumor immunity, with added benefit when combined with PD-1 or PD-L1 blockade [241]. In parallel, Xu et al. demonstrated that ionizable lipid nanoparticles co-delivering mRNA encoding a Kirsten rat sarcoma viral oncogene homolog (KRAS) G12D neoepitope together with the STING agonist cGAMP reprogrammed an immunotolerant metastatic niche, induced IFN-driven APC activation and cytotoxic T-cell responses, suppressed metastatic growth, and prolonged survival in a metastatic pancreatic cancer model, providing a mechanistic template for pairing neoantigen-encoding mRNA with innate immune agonists in metastatic disease [242].

Overall, the most convincing evidence discussed in this section is bone-metastasis-specific but preclinical, showing that nanoparticle-enabled siRNA delivery, mRNA-based immune activation, phototherapy-based immune priming, and checkpoint-sensitizing designs can reduce skeletal tumor burden, limit osteolysis, or enhance local antitumor immunity in experimental models of bone metastasis [224,232,234]. However, these findings should not be interpreted as clinical validation of nano-immunotherapy for metastatic bone disease, because the supporting data remain largely confined to murine and other preclinical systems [221,222,224,234]. In particular, claims regarding immune reprogramming and synergy with checkpoint blockade are supported mainly by mechanistic and efficacy studies in model systems, whereas direct clinical or near-clinical evidence in patients with bone metastases remains lacking [224,230]. Current clinical relevance is therefore best understood as platform-level precedent for nanomedicine and nucleic-acid delivery rather than bone-metastasis-specific proof of benefit. Consistent with this, bone metastases remain a setting of immune resistance characterized by myeloid suppression, T-cell dysfunction, osteoclast-linked immunoregulation, and organ-level under-response to checkpoint inhibitors, all of which represent likely failure modes that may limit translation unless they are specifically addressed in future therapeutic designs [227,229,230,231,243].

## 5. Synergy with Conventional Therapies

### 5.1. Chemotherapy–Nanoparticle Synergy in Bone Metastasis

Cytotoxic chemotherapy remains a cornerstone of treatment for metastatic breast and prostate cancers with skeletal involvement; however, its immunologic effects are now recognized as being as important as its direct tumor cell killing. In bone metastasis, where immune exclusion, osteoclast-driven TGF-β release, and myeloid dominance converge to blunt adaptive immunity, chemotherapy can act as an immunologic catalyst when paired with rational nanoparticle design.

#### 5.1.1. Immunogenic Cell Death as a Skeletal Immune-Priming Event

Anthracyclines and taxanes induce ICD, marked by calreticulin exposure, HMGB1 release, and ATP secretion, which together promote dendritic cell (DC) recruitment and cross-presentation of tumor antigens [225]. In murine breast cancer models, doxorubicin increases CD8^+^ T-cell infiltration and improves tumor control in a TLR4-dependent manner, demonstrating that cytotoxic injury can initiate adaptive immune priming [207].

In the context of bone metastasis, ICD has additional implications. Tumor cell apoptosis diminishes osteoclast-activating signals (such as RANKL and parathyroid hormone-related protein (PTHrP)), thereby reducing osteolysis and limiting the release of matrix-bound TGF-β—a cytokine central to immune exclusion within skeletal lesions [244]. In 4T1 bone metastasis models, chemotherapy helps couple cytotoxic debulking with bone remodeling and immune activation. As tumor burden falls, osteoclast-activating signals decline, which attenuates trabecular bone loss and constrains TGF-β liberation [244]. This decrease in immunosuppressive cues permits a transient rise in CD8^+^ T-cell infiltration. These activated T-cells then reinforce an anti-osteoclastic milieu by secreting IFNγ and IL-4, further suppressing osteoclastogenesis and stabilizing the skeletal microenvironment [232]. However, these immune gains are often transient due to the rapid re-establishment of suppressive circuits.

#### 5.1.2. Chemotherapy-Induced PD-L1 Adaptation and Checkpoint Escape

A paradoxical effect of chemotherapy is the induction of adaptive checkpoint resistance. Doxorubicin and docetaxel upregulate PD-L1 expression on tumor cells through NF-κB- and IFN-γ-dependent signaling cascades [245]. In breast cancer models, chemotherapy-induced PD-L1 elevation reduced CD8^+^ T-cell cytotoxicity but was reversed by PD-1/PD-L1 blockade, showing that cytotoxic therapy can simultaneously prime and restrain antitumor immunity [245].

This mechanism is especially pertinent in bone metastases, where PD-L1^+^ myeloid populations are already abundant. Single-cell profiling of human prostate cancer bone metastases revealed increased PD-L1 expression on tumor-associated macrophages and monocytes, accompanied by exhaustion signatures in tumor-proximal CTL [231]. Thus, chemotherapy-driven PD-L1 upregulation within an already suppressive marrow niche may intensify immune dysfunction unless checkpoint pathways are concurrently neutralized.

Nanoparticle platforms that co-deliver chemotherapy with PD-L1 siRNA or checkpoint inhibitors seek to counteract this adaptation. In bone-targeted lipid nanoparticles carrying docetaxel and PD-L1 siRNA, PD-L1 knockdown increased CD8^+^ T-cell infiltration and reduced skeletal tumor burden compared with docetaxel alone [245]. Mechanistically, these systems prevent therapy-induced checkpoint reinforcement while preserving ICD-mediated priming.

#### 5.1.3. Myeloid Reprogramming and Osteoclast–Immune Coupling

Bone metastases are enriched for myeloid-derived suppressor cells (MDSCs) and M2-like macrophages that suppress cytotoxic T-cells and promote osteoclastogenesis [232,246,247]. In 4T1 bone metastasis, MDSCs accumulate within skeletal lesions and express high levels of PD-L1, directly impairing T-cell effector function [232]. Moreover, suppressed T-cells in bone can adopt pro-osteoclastogenic phenotypes via TNF-α and RANKL production, reinforcing osteolysis and tumor progression [248].

Systemic delivery of paclitaxel via ATP-conjugated polymeric nanoparticles overcomes the immunosuppressive microenvironment by inducing immunogenic cell death and providing a stable ‘find-me’ signal. This formulation enhances the recruitment of dendritic cells and activates CD8^+^ T-cell immunity more effectively than free drugs, leading to superior tumor suppression [249].

Additional platforms combining chemotherapy with STAT3 inhibition or STING agonists have shown enhanced IL-12 production, reduced arginase-1 expression, and improved T-cell-dependent tumor control in metastatic models, suggesting that coordinated targeting of tumor cells and suppressive myeloid compartments is essential for durable skeletal immune restoration [250].

#### 5.1.4. Bone-Targeted Co-Delivery and Therapeutic-Index Optimization

Systemic chemotherapy is frequently limited by myelosuppression, which is particularly problematic in patients with extensive skeletal disease. Bone-targeting strategies, such as bisphosphonate-functionalized nanoparticles, enhance drug retention within mineralized lesions while reducing systemic exposure [219].

In prostate cancer bone metastasis models, alendronate-modified nanocarriers co-delivering docetaxel achieved superior tumor suppression and preservation of trabecular architecture compared with free docetaxel, accompanied by reduced osteoclast numbers and increased CD8^+^ T-cell infiltration [27,219]. These findings indicate that bone-targeted co-delivery not only enhances cytotoxic efficacy but also interrupts tumor–osteoclast–immune coupling.

Collectively, chemotherapy in bone metastasis should be reframed as a programmable immunologic catalyst. When integrated into nanoparticle systems that suppress PD-L1 adaptation, reprogram myeloid suppression, and concentrate drug within skeletal lesions, chemotherapy can amplify antigen release, dismantle osteoimmune resistance circuits, and improve therapeutic index in the marrow microenvironment.

### 5.2. Bone-Modifying Agents and Nanoparticles

Bone-modifying agents (BMAs), such as bisphosphonates and RANKL inhibitors, remain standard of care for skeletal metastases, primarily to prevent skeletal-related events. However, emerging data indicate that osteoclast-targeted therapies also exert immunologic and tumor-modulatory effects that extend beyond bone stabilization. Because osteoclast activity drives TGF-β release, immune suppression, and tumor progression within the bone microenvironment, nanoparticle-enabled delivery of BMAs or osteoclast-modulating agents offers an opportunity to mechanistically disrupt the tumor–bone “vicious cycle” while improving therapeutic precision. Although many principles of nanomedicine apply across solid tumors, bone metastasis offers distinct advantages for nanoparticle design. Hydroxyapatite-rich surfaces support bone-selective targeting, while acidic and protease-rich resorption sites allow localized drug release. In addition, bone lesions depend on osteoclast-linked immunosuppressive pathways, creating therapeutic targets that are especially relevant in the skeleton. As a result, nanoparticle therapy in bone metastasis aims not only to boost antitumor immunity but also to limit osteolysis and preserve bone structure.

#### 5.2.1. Osteoclast Inhibition and Interruption of the Tumor–Bone Vicious Cycle

Osteoclast-mediated bone resorption releases matrix-stored growth factors, including TGF-β, which promotes tumor proliferation, immune exclusion, and further osteoclast activation, creating a feed-forward loop in skeletal metastasis [251]. While Zoledronic acid consistently reduces osteolytic lesions and niche signaling (e.g., Dkk1 or TGF-β), its impact on absolute tumor burden can vary by model, with some prostate cancer studies showing that tumor growth can persist even when bone resorption is inhibited [252].

Beyond structural preservation, osteoclast inhibition has immunologic consequences. TGF-β suppression restores CD8^+^ T-cell infiltration and enhances responsiveness to immune checkpoint blockade in TGF-β-rich metastatic models [252]. These findings suggest that BMAs may indirectly sensitize bone lesions to immunotherapy by modulating cytokine gradients and stromal architecture. However, systemic bisphosphonate exposure is limited by renal toxicity and suboptimal accumulation within tumor-adjacent bone surfaces.

Nanoparticle encapsulation of bisphosphonates enhances skeletal targeting. Alendronate-functionalized nanocarriers demonstrate preferential accumulation in hydroxyapatite-rich metastatic bone and improved suppression of osteoclast activity compared with free drugs [219]. In prostate cancer bone metastasis models, alendronate-modified nanoparticles reduced tumor-associated osteoclast numbers and attenuated trabecular bone loss while improving local drug retention [219]. Mechanistically, these systems spatially concentrate osteoclast inhibition at sites of active resorption, interrupting growth-factor release that fuels tumor progression.

#### 5.2.2. RANKL Pathway Targeting and Immune Modulation

RANKL signaling is central to osteoclast differentiation and survival. Therapeutic inhibition of RANKL with monoclonal antibodies such as denosumab reduces skeletal-related events in metastatic cancer. Preclinical models further suggest that RANKL blockade can reshape immune composition within the bone microenvironment. In particular, RANKL signaling promotes the expansion of suppressive myeloid populations and supports the survival of tumor-associated macrophages [253].

In murine bone metastasis, genetic or pharmacologic inhibition of RANKL reduced osteoclast numbers, decreased tumor burden, and was associated with increased CD8^+^ T-cell infiltration [253]. These findings directly connect osteoclast biology to the regulation of adaptive immunity. However, systemic RANKL inhibition alone may be insufficient to fully reverse immune suppression within established lesions.

Nanoparticle-based strategies that co-deliver RANKL inhibitors with immunomodulatory agents are emerging. Hydroxyapatite-binding nanoparticles carrying RANKL siRNA or decoy peptides have demonstrated enhanced localization to bone surfaces and superior suppression of osteolysis relative to free inhibitors in metastatic models [27]. By combining RANKL blockade with localized immune-activating modules, these platforms aim to simultaneously disrupt osteoclastogenesis and restore antitumor immunity within skeletal lesions.

#### 5.2.3. Targeting TGF-β and Osteoclast-Linked Immune Checkpoints

TGF-β is a central driver of immune exclusion in bone metastasis, a process further reinforced by osteoclast-mediated matrix degradation [251]. In preclinical metastatic models, pharmacologic inhibition of TGF-β receptor signaling restored T-cell infiltration and improved responses to PD-L1 blockade [254]. Nanovesicles co-delivering TGF-β receptor inhibitors have been shown to enhance cytotoxic T-cell infiltration into tumors and to improve therapeutic synergy with checkpoint blockade [255].

Recent work further identifies osteoclast-lineage immune checkpoints, such as Siglec-15, as modulators of T-cell suppression in bone metastasis. Siglec-15 is upregulated in the bone metastatic niche and promotes osteoclastogenesis while suppressing CD8^+^ T-cell activation. In murine breast cancer bone metastasis models, Siglec-15 neutralization reduced osteolysis and improved survival, indicating that osteoclast-targeted immune checkpoints represent dual regulators of bone destruction and immune suppression [233].

Biomaterial-based local delivery systems integrating Siglec-15 targeting with chemotherapeutic or immunomodulatory payloads have demonstrated reduced tumor burden and decreased bone destruction in skeletal metastasis models [256]. Mechanistically, these constructs couple the inhibition of osteoclast-mediated suppression with cytotoxic priming, addressing both structural and immune components of the metastatic niche.

#### 5.2.4. Therapeutic-Index Optimization Through Bone-Targeted Nanocarriers

Conventional BMAs are administered systemically and distributed broadly to areas of active bone turnover, including non-malignant skeletal sites. In contrast, nanoparticle engineering enables more selective accumulation at tumor-adjacent bone surfaces by exploiting bisphosphonate–hydroxyapatite interactions or calcium-binding moieties [219]. Such targeting increases intralesional drug concentration while reducing systemic exposure, potentially mitigating nephrotoxicity and hypocalcemia risks associated with high-dose bisphosphonates.

In prostate and breast cancer bone metastasis models, bone-targeting nanocarriers delivering osteoclast inhibitors achieved superior preservation of trabecular architecture and reduced tumor volume compared with free agents [233,256]. Importantly, osteoclast suppression within these systems was accompanied by improved CD8^+^ T-cell infiltration, supporting the concept that structural bone stabilization and immune restoration are mechanistically coupled processes.

Collectively, nanoparticle-enabled bone-modifying strategies extend beyond the prevention of skeletal-related events. By spatially concentrating osteoclast inhibition, attenuating TGF-β release, targeting osteoclast-linked immune checkpoints, and integrating immune co-therapies, these platforms disrupt the tumor–bone vicious cycle while enhancing immune responsiveness within the marrow microenvironment.

### 5.3. Emerging Multi-Modal Nanoparticle Platforms: Integrative Disruption of the Bone Metastatic Niche

Single-axis interventions in bone metastasis rarely produce durable control because tumor progression is sustained by tightly coupled circuits linking osteoclast activation, immune suppression, matrix-derived growth factors, and adaptive checkpoint resistance. Emerging multi-modal nanoparticle platforms aim to simultaneously target tumor cells, osteoclasts, and immune checkpoints within skeletal lesions, thereby collapsing the tumor–bone vicious cycle while improving therapeutic index.

#### 5.3.1. Chemo-Immuno-Osteoclast Integrated Platforms

Recent bone-targeted platforms integrate cytotoxic therapy, checkpoint modulation, and osteoclast pathway suppression within a single construct. In a tibial breast cancer bone metastasis model, a transformable supramolecular nanoparticle that combined photodynamic therapy (PDT) with IDO-1 inhibition synergized with anti-PD-1 treatment, leading to pronounced increases in intratumoral CD8^+^ T-cell infiltration and a significant reduction in osteolytic burden compared with the respective monotherapies [224]. This design mechanistically couples ICD-driven antigen release with the metabolic relief of T-cell suppression and checkpoint blockade.

Similarly, nanoparticle systems co-delivering docetaxel and PD-L1 siRNA with alendronate-mediated bone targeting achieved greater tumor suppression and reduced osteoclast numbers in prostate cancer bone metastasis models compared with free docetaxel [27]. By preventing chemotherapy-induced PD-L1 adaptation while concentrating cytotoxic exposure at resorptive bone surfaces, these platforms synchronize tumor debulking with immune restoration and structural stabilization. These integrated chemo-immuno-osteoclast therapeutic interactions are summarized in Figure 2.

#### 5.3.2. Targeting Osteoimmune Checkpoints and Systemic Bone-Derived Signals

Emerging evidence indicates that bone metastases can exert systemic immunosuppressive effects. In a 2025 mechanistic study, osteoclast-derived osteopontin (OPN) produced within bone metastases impaired CD8^+^ T-cell recruitment and precursor differentiation at extraosseous tumor sites, diminishing checkpoint blockade efficacy; targeting osteoclasts or the OPN axis restored responsiveness to immune therapy [230]. These findings underscore how bone lesions can exert an endocrine-like influence on systemic antitumor immunity.

Parallel work has identified Siglec-15 as an osteoclast-associated immune checkpoint that is enriched within bone metastatic niches. Osteoclast-derived apoptotic bodies were shown to suppress naïve CD8^+^ T-cell activation through Siglec-15-dependent mechanisms, and Siglec-15 blockade reduced secondary metastases and improved survival in murine breast cancer bone metastasis models [233].

Multi-modal biomaterial systems that combine Siglec-15 targeting with localized chemotherapy or photothermal activation have been shown to reduce osteolysis, diminish suppressive myeloid populations, and enhance cytotoxic T-cell infiltration within bone lesions [256]. These platforms mechanistically converge on dual suppression of osteoclast-driven immune inhibition and tumor growth.

#### 5.3.3. TGF-β Modulation and Microenvironmental Reprogramming

Because osteoclast-mediated bone resorption releases matrix-bound TGF-β, which in turn reinforces immune exclusion and tumor growth, multi-modal platforms that incorporate TGF-β pathway inhibition are of particular interest. In preclinical models, combined blockade of TGF-β signaling and PD-L1 restored T-cell infiltration and improved tumor control in immune-excluded settings [244]. Nanovesicle-based systems delivering TGF-β receptor inhibitors alongside immune-activating components have enhanced cytotoxic T-cell penetration and therapeutic response compared with monotherapy [255].

When adapted to bone-targeted formats, such strategies offer the potential to simultaneously suppress TGF-β-driven stromal exclusion, reduce osteoclastogenesis, and enhance checkpoint responsiveness. Conceptually, this integrates structural niche stabilization with immune reactivation, addressing both mechanical and immunologic determinants of skeletal disease progression.

#### 5.3.4. Systems-Level Therapeutic Index Optimization

Multi-modal nanoparticle constructs also allow for tighter spatial and temporal control of therapy. Bone-homing ligands, such as bisphosphonate modifications, concentrate payload delivery at mineralized metastatic surfaces, increasing local drug exposure while limiting systemic toxicity [54]. In preclinical bone metastasis models, bone-targeted nanocarriers delivering combined immunomodulatory and cytotoxic agents achieved superior preservation of trabecular architecture and greater CD8^+^ T-cell infiltration than free-drug combinations [54].

Collectively, these emerging multi-modal platforms move the field beyond simple drug add-ons toward coordinated reprogramming of the bone metastatic niche. By combining chemotherapy, checkpoint modulation, osteoclast inhibition, and immune priming within a single bone-targeted system, they seek to disrupt the tumor–bone vicious cycle at multiple points while improving the therapeutic index in the marrow microenvironment.

## 6. Safety, Pharmacokinetics, and Translational Barriers

Taking immunomodulatory nanoparticles from the bench to the clinic requires overcoming multiple challenges that can be categorized into biological, engineering, and regulatory barriers [257]. Among these, biological barriers, particularly the profound heterogeneity of bone lesions and the delicate osteoimmunological balance of the bone marrow, are arguably the most limiting. Even with perfectly scaled manufacturing, therapies will fail if they cannot penetrate the sclerotix matrix or if they trigger dose-limiting systemic immune toxicity. Potential solutions to these biological hurdles include active bone-targeting ligands and stimuli-responsive designs to enhance local accumulation. Meanwhile, engineering and regulatory barriers are increasingly being addressed by the adoption of continuous microfluidic manufacturing, Quality by Design (QbD) protocols, and the drive for standard global safety evaluation frameworks.

### 6.1. Biological Barriers: Safety, Immune Trade-Offs, Osteoimmunology, and Tumor Heterogeneity

The systemic administration of nanoparticles carries the risk of immune hyperactivation or immunosuppression, as the immune system reacts to the size, surface charge, and composition of the nanoparticles [258,259]. Notably, intravenous infusion can trigger complement activation-related pseudoallergy and complement activation-related pseudoallergy (CARPA), which is a rapid, non-IgE-mediated reaction. In CARPA, the complement cascade is activated as anaphylatoxins (C3a and C5a) are released. This causes mast cells and basophils to degranulate, potentially leading to severe reactions, such as anaphylaxis [260].

Potent immune agonists (e.g., TLR or STING agonists) can also precipitate systemic inflammatory response syndrome (SIRS) [259]. Activated macrophages and dendritic cells in response to these agonists release massive quantities of pro-inflammatory cytokines (IL-1β, TNF-α, IL-6, IL-12). The strength of this response depends critically on degradation kinetics. With rapidly degrading materials like poly(alkyl cyanoacrylate), exposure to immune cells is minimized. In contrast, inorganic materials that degrade poorly will accumulate in tissues. This can stimulate the production of ROS chronically and cause tissue necrosis [258].

As nanoparticles circulate, they acquire a protein “corona” made of plasma proteins, lipids, and sugars. Proteins in this corona, such as C3b, opsonize the particles, which marks them for clearance by the reticuloendothelial system (RES) in the liver and spleen. This off-target uptake by macrophages shunts the dose away from the bone metastases and also poses a risk of organ toxicity [260]. Engineering the surface of nanoparticles can help mitigate this RES clearance. While traditional PEGylation can prolong circulation, over-PEGylation can hinder tumor uptake and induce accelerated clearance from the blood. Biomimetic cloaking is another technique. It uses cell membranes from cancer or immune cells to confer natural immune evasion. In addition, maintaining particle diameters between 50 and 150 nm and neutralizing surface charge independently promotes bone marrow accumulation [261].

Another significant barrier is off-target immune activation. When immune adjuvants like high-dose IL-2 or IL-12 are delivered systemically, there is a risk of cytokine release syndrome and multi-organ damage [172]. In the bone marrow, the same mechanisms that recruit immune cells, such as modifying cytokine profiles or activating macrophages, also carry the risk of bone marrow toxicity and hematopoietic disruption. The hematopoietic stem cells and progenitor cells are also vulnerable to nanoparticles, which can potentially lead to immunosuppression or disrupted hematopoiesis. This creates a delicate osteoimmunological balance, because while the aim of the treatments is to break the osteolytic cycle and reverse immune suppression, normal skeletal remodeling as well as hematopoiesis can also be impaired [53]. Together, these immune interactions and clearance pathways influence nanoparticle pharmacokinetics, ultimately determining what fraction of the administered dose reaches the bone lesions.

Even if nanoparticles can evade systemic clearance, their accumulation in bone metastases is limited by both interpatient and intratumoral heterogeneity. In bone lesions, elevated interstitial fluid pressure hinders passive diffusion, blocking the extravasation of nanoparticles into the tumor core [257].

Because of this architectural variability, nanoparticle accumulation has been reported to differ by up to 35-fold among patients, independent of systemic plasma exposure. This uneven drug distribution can cause pseudo-resistance, meaning treatments can fail because of sub-therapeutic concentrations reaching the bone lesions [258]. Overcoming this pseudo-resistance requires strategies to actively target the metastases in the bones. For instance, bisphosphonates bind directly to the hydroxyapatite matrix in bone, promoting particle accumulation. Another example is superparamagnetic iron oxide nanoparticles (SPIONs) coupled with external magnetic fields, which mechanically pull the therapeutic into dense sclerotic lesions, thereby mitigating high interstitial fluid pressure [259]. Physiologically based pharmacokinetic modeling can help predict patient-specific variability in particle delivery [260].

### 6.2. Engineering Barriers: Manufacturing and Stability Challenges

The production of these therapies is a major engineering challenge. Nanomedicines are classified as non-biological complex drugs (NBCDs), meaning their safety and efficacy depend on precise characteristics. These include size, narrow polydispersity, surface charge, and exact spatial orientation of surface modifications, which must all be maintained under Good Manufacturing Practice conditions [257]. When traditional methods of bulk synthesis are scaled up, reaction kinetics and fluid mechanics change. These altered dynamics can lead to severe batch variability, including broad size distributions and premature particle aggregation [261]. The integration of multiple functional components, such as targeting ligands and immunomodulators, can further exacerbate this variability, making large-scale production with consistent quality challenging and costly.

Regulatory authorities now mandate a Quality by Design (QbD) framework during scale-up [257]. This requires rigorous analytical characterization to define the nanoparticle’s Critical Quality Attributes and to establish strict release specifications to ensure that every batch meets predefined safety and efficacy benchmarks. Furthermore, establishing appropriate sterilization procedures that do not degrade delicate biological components or trigger premature payload release is a significant hurdle for clinical translation. Under QbD principles, manufacturers are shifting to continuous microfluidic assembly and 3D hydrodynamic flow focusing. These technologies tightly control the rapid mixing of organic and aqueous phases, helping make the structures uniform [262].

Even when particles are synthesized perfectly, long-term stability still poses an issue. Conjugating ligands such as antibodies or bone-homing peptides makes the structure fragile, requiring precise ligand-density control to ensure consistent targeting capabilities. Storing these formulations in aqueous suspensions for long durations frequently leads to shelf-life instability, which manifests as ligand detachment, hydrolytic degradation of the polymer backbone, payload leakage, and particle agglomeration [257]. Commercial viability, therefore, requires advanced conjugation techniques and lyophilization protocols to ensure a stable global shelf life [263].

### 6.3. Regulatory Barriers: Standardization and Clinical Evaluation

Fragmented regulations in different regions present another challenge. A fundamental obstacle is that there is no universal size threshold or definition of a “nanomaterial” among authorities such as the United States Food and Drug Administration and the European Medicines Agency. This lack of standardization increases the financial burden of obtaining clinical approval across international markets [264]. In addition, immunomodulatory nanoparticles for bone metastases are often classified as combination products, as a single carrier can contain a small-molecule drug, a biological targeting antibody, and an inorganic core [102].

Regulation of such combination products depends on the Primary Mode of Action (PMOA), the action that contributes most to the effect. However, establishing a single PMOA can be challenging because these particles rely on synergy among components. This ambiguity can trigger disputes over jurisdiction and overlapping reviews between different centers, resulting in complex approval pathways. Furthermore, manufacturers must provide specialized preclinical safety data, including long-term biodistribution and biodegradation studies. This data must show that synthetic polymers or inorganic materials do not induce delayed immunotoxicity, granuloma formation, or chronic organ damage [102].

### 6.4. Current Clinical Landscape and Translational Outlook

A critical barrier in the field is the continued lack of bone-specific clinical validation for complex immunomodulatory nanocarriers. Currently, the majority of evidence on bone-targeted nanoparticle immunotherapy, such as STING-agonist platforms, engineered macrophages, and biomimetic camouflage, remains confined to preclinical models. However, clinical studies and trials in adjacent areas help inform the current translational landscape. Clinical data on the conventional use of systemic immune checkpoint inhibitors establishes the baseline necessity for immunomodulatory nanoparticles, with bone involvement recognized as an independent adverse prognostic factor that renders the skeletal niche immunologically cold and resistant to standard care [47,265].

While efficacy data for nanotherapy in human bone metastases are lacking, clinical pharmacokinetic imaging has successfully quantified the severe delivery barriers. PET trials utilizing radiolabeled nanoparticles have demonstrated up to a 35-fold interpatient variability in nanoparticle accumulation at metastatic sites, independent of systemic plasma exposure. Clinical data show that human bone lesions exhibit profound architectural heterogeneity that limits passive delivery. Further, while data on secondary bone metastasis is scarce, the ongoing clinical evaluation of localized physical treatments such as superparamagnetic iron oxide nanoparticles (SPIONs) for magnetic hyperthermia in primary bone tumors, provides a crucial safety and feasibility precedent for penetrating dense sclerotic bone lesions in humans [266,267].

Ultimately, translating these nanotherapies will require bridging the gap between these established physical delivery platforms and the sophisticated immune-reprogramming payloads currently under investigation in preclinical models.

## 7. Preclinical Models and Evaluation

For the preclinical evaluation of immunomodulatory nanoparticles, specialized models that can mimic the interactions between cancer cells, immune cells, and the bone microenvironment are required. Conventional subcutaneous xenograft models are inadequate because they lack the unique architecture and cellular environment of the bone [268]. Instead, researchers use alternatives such as immunocompetent syngeneic models, humanized mice, and advanced ex vivo platforms.

### 7.1. Immune-Competent Models

To test immunomodulatory therapies, a host with a fully functional immune system is required [269]. The standard starting point is syngeneic mouse models, in which murine cancer cells are injected into genetically identical mice. For example, the 4T1 breast cancer model in BALB/c mice is often used to study aggressive osteolytic bone metastases [270]. Intratibial or intracardiac injections are often utilized so that isolated bone lesions can be created quickly, and the animal is not overwhelmed with lung or liver tumors [264].

On the other hand, the RM1 and PC3 models in C57BL/6 mice help simulate the mixed or osteoblastic lesions characteristic of prostate cancer [269]. These models can be useful for evaluating how nanoparticles affect osteoblast hyperactivation and the reprogramming of the local immune system. However, they still have major limitations, including differences from humans in immune cell markers, cytokine profiles, and MHC presentation.

### 7.2. Humanized Mouse Models

To help bridge the gap between mouse and human immunology, humanized mouse models can be used. In these models, severely immunodeficient hosts like non-obese diabetic/severe combined immunodeficient (NOD/SCID) or NOD scid gamma (NSG) mice are engrafted with human peripheral blood mononuclear cells (PBMCs) or hematopoietic stem cells. These humanized models are important for evaluating nanoparticles that specifically target human immune components, such as humanized antibodies for immune checkpoint inhibition (e.g., anti-PD-1) or CRISPR guide RNAs. However, although these models are useful, they are technically intensive and expensive to use. Another limitation is that they have a short window for evaluation because these mice eventually develop graft-versus-host disease [263,271].

### 7.3. Bone Organoids and Ex Vivo Systems

Animal models present ethical constraints and physiological differences from humans, which have spurred the development of 3D organoids [268]. In bone organoids, biocompatible scaffolds (such as hydrogels or degradable polymers) are often 3D-printed to mimic the porous structure of cancellous bone. Inside these matrices, osteoblasts, osteoclasts, patient-derived cancer cells, and immune cells can be co-cultured to build an integrated ecosystem [272,273]. Advanced organ-on-a-chip models also incorporate microfluidic channels to simulate vascular perfusion and interstitial fluid pressure. This allows for evaluating how nanoparticles penetrate bone lesions and how immune cells are recruited under physiological conditions [274]. Thus, bone organoids and ex vivo systems are also useful for mechanistic evaluation of immunomodulation by nanoparticles.

### 7.4. Translational Limitations of Current Preclinical Models

While murine models have been foundational for evaluating nanoparticle immunotherapies, there are also significant species-to-species differences in the bone marrow niche and the clinical translation potential of these models. The immune and cellular systems of mice, as well as other species, differ fundamentally from those of humans, including differences in immune cell markers, cytokine networks and interplay, MHC presentation, and tumor-stromal interactions. Because of inherent differences in immune response between, for example, human and mouse immune responses, overreliance on such preclinical models can lead to inappropriate assessment of therapeutic efficacy. The biological differences also affect pharmacokinetics, dosing, and optimal treatment timing parameters. Thus, other models and systems, such as ex vivo analyses, have an important role in predicting human responses before proceeding to clinical trials [272,275].

### 7.5. Evaluation Endpoints

Evaluating the success of nanoparticle treatment requires tracking its distribution, quantifying its effect on bone structure, and measuring its impact on the immune system.

Tracking the distribution of nanoparticles in living animals relies primarily on various non-invasive imaging modalities. PET uses radioactive isotopes like ^64^Cu or ^89^Zr to quantify the percentage of the injected dose that reaches bone metastases [258,275]. Magnetic resonance imaging (MRI) is ideal for tracking particles that contain, for example, superparamagnetic iron oxide. Finally, near-infrared (NIR) fluorescence is frequently used to visualize the accumulation of carriers in shallow bone beds [276].

Next, to quantify the effect of nanoparticles on bone destruction, high-resolution micro-computed tomography (µCT) is the gold standard. Micro-CT measures indices like trabecular bone volume fraction (BV/TV) and cortical porosity to objectively delineate osteolysis and osteoblastic overgrowth [276].

The ultimate goal is to transform the tumor microenvironment from a “cold” immunosuppressive state to an immunologically “hot”, reactive state. To measure this shift, cellular and molecular profiling techniques must be used. Flow cytometry and multiplex immunohistochemistry quantify the density of CD8+ cytotoxic T-cells and also determine whether these cells are actively proliferating, or if they are expressing exhaustion markers (e.g., PD-1, TIM-3) [277]. Another key metric is macrophage polarization, given their dominance in the bone microenvironment. This can be assessed by looking for a shift from the tissue repair M2 phenotype (e.g., CD206, CD162) to the pro-inflammatory anti-tumor M1 phenotype (e.g., CD86, MHC-II). Finally, cytokine and chemokine profiling also helps confirm successful immune remodeling. A decrease in suppressive cytokines such as TGF-β and RANKL and an increase in stimulatory factors like IFN-γ and IL-12 are positive indicators [278,279].

## 8. Conclusions and Future Directions

Immunomodulatory nanomedicine is a next-generation strategy that represents a paradigm shift in the treatment of bone metastases. Current treatments, such as chemotherapy and palliative radiation, can fail to eradicate the metastases and can cause severe toxicity [276]. Even immune checkpoint inhibitors show poor outcomes in patients who have bone involvement, because the bone acts as an immunologically “cold” sanctuary for cancer, with factors like TGF-β and myeloid suppressor cells contributing to this phenomenon [275]. By actively delivering their payloads directly to the bone lesions, nanoparticles can potentially disrupt the cycle of bone breakdown and reprogram the immune system without inducing systemic immune toxicity [272].

Moving forward, the field of nanomedicine is positioned to benefit from personalized medicine technologies such as nanovaccines. Traditional tumor vaccines often fail because of central immune tolerance, but emerging treatments can exploit the unique set of mutations in an individual patient’s tumor. Advanced algorithms can identify patient-specific neoantigens, which can then be engineered into stable nanoparticles along with immune adjuvants [280]. Such systems can drive robust cross-presentation, priming a durable CD8+ cytotoxic T-cell response capable of targeting dormant tumor cells in the skeleton [277]. In addition to these personalized vaccines, genetic engineering CRISPR/Cas9 nano-systems are also being developed to knock down genes that drive metastasis. Optimized nanocarriers can evade rapid degradation by nucleases, which usually hinders the systemic delivery of CRISPR. This will allow for deleting critical immune checkpoints such as PD-L1 from cancer cells, knocking down their immune-suppressing capability [35].

To further improve payload delivery, biomimetic cell membrane-coated nanoparticles and exosomes are being developed. They can naturally evade phagocytosis and avoid acquiring a protein corona [281]. These biomimetics naturally home in to pre-metastatic sites, allowing for immunotherapeutics to be delivered. This can prophylactically disrupt immunosuppression in the bone microenvironment before tumor cells can overtly colonize it [282]. To maximize drug capacity and stability under high interstitial fluid pressure, rigid inorganic cores, such as mesoporous silica, are being developed for these organic cloaks. These hybrid nanoparticles combine the biocompatibility of natural cell membranes with the stability of inorganic materials [283].

Ultimately, clinical implementation of such therapies will require specially designed clinical trials. Current randomized controlled trials rarely stratify patients by bone involvement, resulting in limited data for this cohort. Future trials must, therefore, explicitly recognize the bone as a distinct immune compartment [275]. Since immunotherapy with single agents often fails to overcome the bone tumor microenvironment, trials should evaluate the synergy of bone-targeting nanoparticles with systemic immune checkpoint inhibitors and bone-modifying agents. The goal is to use nanomedicine to “warm” the tumor microenvironment from the inside, making resistant bone metastases susceptible to systemic immunotherapy and resulting in a durable clinical response [272].

## Figures and Tables

**Table 1:** Translational precedent of major nanoparticle platform classes in oncology and their potential relevance to bone metastasis immunotherapy.

Platform Class	Representative Clinical Precedent	Potential Relevance to Bone Metastasis Immunotherapy	Main Limitation
Liposomal nanomedicines	Pallares and Abergel, 2025 [146]	Shows the strongest clinical precedent for nanocarrier translation in oncology, including metastatic disease	Most approved liposomal systems are not bone-targeted and were not developed for marrow immune reprogramming
Polymeric nanomedicines	Pallares and Abergel, 2025 [146]	Provides translational precedent for polymer-based nanomedicines, a platform class widely used for experimental targeted and nucleic acid delivery systems	Bone-metastasis-specific immune applications remain largely preclinical, and clinical success is less mature than for liposomal systems
Iron oxide nanoparticles	Maier-Hauff et al., 2010 [147]	Provides clinical proof-of-concept that magnetic iron-oxide nanoparticles can be safely translated as hyperthermia-enabled therapeutic platforms in oncology	Clinical evidence is localized and indication-specific (glioblastoma), not bone-metastasis-specific and not designed for marrow immune reprogramming
Hafnium oxide nanoparticles (radioenhancers)	Bonvalot et al., 2019 [148]	Provides randomized clinical proof-of-concept that inorganic nanoparticles can potentiate local radiotherapy in solid tumors, supporting the translational feasibility of nanoparticle-enabled combination strategies	Evaluated as intratumoral radioenhancers in soft-tissue sarcoma rather than as bone-targeted or immunomodulatory nanotherapies for metastatic bone disease

**Table 2:** Representative nanoparticle platforms, quantitative outcomes, and comparator conditions from selected bone-targeted studies.

Reference	Platform/Payload	Model	Quantitative Outcome	Comparator/Reference Condition	Key Implication
Chaudhari et al. [155].	Zoledronate-conjugated PLGA-PEG nanoparticles loaded with docetaxel	Breast cancer bone metastasis model	Particle size 132 ± 9.5 nm; drug entrapment 73.53 ± 3.43%; 5.51% drug loading; prolonged blood-circulation half-life, reduced liver uptake, and significantly higher bone-site retention with enhanced tumor retention	Compared with non-targeted pegylated PLGA nanoparticles (PLGA-PEG NPs); in vitro activity also compared across other tested formulations	Zoledronate conjugation improved bone localization and retention of docetaxel-loaded nanocarriers
Wu et al. [156].	ALN-conjugated bone-targeting lipid–polymer hybrid nanoparticles co-encapsulating decitabine (DAC) and arsenic trioxide (ATO)	MDS mouse model	Bone accumulation increased 6.7-fold for DAC and 7.9-fold for ATO versus untargeted nanoparticles at 24 h; plasma exposure increased (AUC_0_–∞: DAC 8685.15 vs. 1932.56 h·mg/L; ATO 4132.46 vs. 1243.40 h·mg/L, BTNPs vs. free drugs); circulation was sustained up to 72 h	Bone accumulation compared with untargeted nanoparticles; pharmacokinetic AUC compared with free DAC and free ATO solutions	ALN-mediated bone marrow targeting improved marrow drug enrichment and exposure, supporting more effective and less toxic MDS therapy
Gao et al. [50].	Dual-ligand P123 polymeric micelles decorated with alendronate (ALN) and DP-8 and loaded with doxorubicin (DOX)	Breast cancer bone metastasis models (3D bone metastasis model and intratibial nude mouse model)	Mean particle size 122.97 ± 4.72 nm; drug loading 3.44%; encapsulation efficiency 76.87% ± 9.72%; HA binding reached ~38% at 30 min and ~52% at 90 min; IC_50_ decreased from 4.69 μg/mL (free DOX) to 0.989 μg/mL (P123-ALN/DP-8@DOX).	Compared with free DOX and non-targeted P123@DOX micelles	Dual bone/tumor targeting improved bone affinity and in vitro antitumor activity, with in vivo enrichment at the bone tumor region and reduced systemic toxicity in a breast cancer bone metastasis model
Mushtaq et al. [157].	mPEG-CHI-siRNA and ALD-PEG-CHI-siRNA nanoparticles	4T1 breast cancer cells (in vitro)	Mean particle size 40 ± 5 nm (mPEG-CHI-siRNA) and 60 ± 5 nm (ALD-PEG-CHI-siRNA); ζ-potential +3 ± 2 mV and +1 ± 1 mV, respectively; serum protection up to 6 h and 4 h, respectively, versus immediate degradation of naked siRNA; at 24 h, remaining wound area was 80% and 42% at 50 nM twist1-siRNA, versus 37% and 6% in untreated controls from the respective experiments	Compared with naked twist1-siRNA and untreated control cells	These nanoparticles achieved efficient twist1-siRNA encapsulation, improved serum stability, reduced TWIST1 protein expression, and delayed 4T1 cell migration, supporting preclinical in vitro potential for bone-metastatic breast cancer gene silencing
Li et al. [158].	Bone-targeting bioreducible polymer vector ALN-Pabol/miRNA polyplex	Murine breast cancer bone metastasis model	Hydroxyapatite binding 91.1%; tumor weight reduced 79.1%; near-complete restoration of bone structure by micro-CT	Tumor weight reduction compared with PBS; additional comparison versus LNP/mRNA showed 36.8% greater reduction	Bone-targeted mRNA delivery produced strong antitumor and anti-osteolytic effects
Florian et al. [47].	Polymeric STING-activating nanoparticles (STING-NPs) loaded with 2′3′-cGAMP	Murine 4T1-592 intratibial breast cancer bone metastasis model	STING-NPs reduced tumor burden at day 7, decreased osteolytic lesion area at days 7 and 14, and fluorescent nanoparticles showed preferential accumulation in tumor-bearing tibiae with 4.2-fold, 2.1-fold, and 9.5-fold higher signal than contralateral control tibiae at 2, 4, and 24 h, respectively; however, day-14 tumor burden was no longer significantly different, and Tregs were ~3-fold higher in treated marrow at day 14	Compared with PBS-treated tumor-bearing mice; biodistribution compared with contralateral non-tumor tibia	Supports transient, not durable, bone marrow immune reprogramming in bone metastasis.
Zhong et al. [132].	Injectable BP@Gel-CD[SA] hydrogel containing black phosphorus nanosheets (BPNSs) + STING agonist	Murine lung cancer bone metastasis models, including femoral LLC bone tumor/post-curettage recurrence model, bilateral distant-tumor model, and bone-defect repair model	60% complete remission after curettage at 4 weeks; mature bone marrow-derived dendritic cells (BMDCs) 26.6%; intratumoral CD8+CD25+ T-cells 16.84 ± 1.18%; Tregs 11.68 ± 1.34%; distant-tumor CD8+CD25+ T-cells 6.92 ± 1.05% and CD80+CD86+ DCs 23.45 ± 1.7%; significant increases in BV/TV and Tb.N	Compared with Gel-CD, BP@Gel-CD, BP@Gel-CD + NIR, Gel-CD[SA], and BP@Gel-CD[SA]	Preclinical evidence that local PTT + STING agonist hydrogel can reduce recurrence, generate systemic antitumor immunity, and support bone regeneration in bone-metastatic disease

## Data Availability

No new data were created or analyzed in this study. Data sharing is not applicable to this article.

## Abbreviations

**Abbreviation**	**Full Terminology/Definition**
ABC	Accelerated Blood Clearance
ADC	Antibody–Drug Conjugate
ALD/ALN	Alendronate
ALP	Alkaline Phosphatase
AMF	Alternating Magnetic Field
AP-1	Activator Protein 1
APC	Antigen-Presenting Cell
AR	Androgen Receptor
ARG1	Arginase-1
ATO	Arsenic Trioxide
ATP	Adenosine Triphosphate
AUC	Area Under the Curve
BMA(s)	Bone-Modifying Agent(s)
BMDC(s)	Bone Marrow-Derived Dendritic Cell(s)
BMP(s)	Bone Morphogenetic Protein(s)
BP(s)	Bisphosphonate(s)
BPNS(s)	Black Phosphorus Nanosheet(s)
BTNPs	Bone-Targeting Nanoparticles
BV/TV	Bone Volume/Total Volume (Trabecular Bone Volume Fraction)
Tb.N	trabecular number
CARPA	Complement Activation-Related Pseudoallergy
CaP	Calcium Phosphate
CaZol NiM	Calcium–Zoledronate Nanoparticles Embedded in Microparticles
CBD	Collagen-Binding Domain
CCL5	C-C Motif Chemokine Ligand 5
cDC1	Conventional Type 1 Dendritic Cell
cGAMP (2′3′-cGAMP)	Cyclic GMP-AMP
cGAS	Cyclic GMP-AMP Synthase
CHI	Chitosan
CLEC9A	C-Type Lectin Domain Family 9 Member A
CpG	Cytosine-Phosphate-Guanine Oligonucleotide
CRISPR	Clustered Regularly Interspaced Short Palindromic Repeats
CRISPR/Cas9	Clustered Regularly Interspaced Short Palindromic Repeats/CRISPR-Associated Protein 9
CRS	Cytokine Release Syndrome
CRT	Calreticulin
CTL	Cytotoxic T Lymphocyte
CTSK	Cathepsin K
CXCL9	C-X-C Motif Chemokine Ligand 9
CXCL10	C-X-C Motif Chemokine Ligand 10
CXCR4	C-X-C Chemokine Receptor Type 4
CyPA	Cyclophilin A
DAC	Decitabine
DAMP(s)	Damage-Associated Molecular Pattern(s)
DC(s)	Dendritic Cell(s)
Dkk1	Dickkopf-Related Protein 1
DOX	Doxorubicin
ECM	Extracellular Matrix
EMT	Epithelial-to-Mesenchymal Transition
EPR	Enhanced Permeability and Retention
ER	Endoplasmic Reticulum
ET-1	Endothelin-1
FDG	Fluorodeoxyglucose
GM-CSF	Granulocyte-Macrophage Colony-Stimulating Factor
HAP	Hydroxyapatite
HMGB1	High Mobility Group Box 1
HSP(s)	Heat Shock Protein(s)
HSP70	Heat Shock Protein 70
HSPC(s)	Hematopoietic Stem and Progenitor Cell(s)
ICD	Immunogenic Cell Death
ICI(s)	Immune Checkpoint Inhibitor(s)
IDO-1	Indoleamine 2,3-Dioxygenase 1
IFN-β	Interferon-Beta
IFN-γ	Interferon-Gamma
IL-1β	Interleukin-1 Beta
IL-2	Interleukin-2
IL-4	Interleukin-4
IL-6	Interleukin-6
IL-10	Interleukin-10
IL-12	Interleukin-12
IL-27	Interleukin-27
IL-30	Interleukin-30
IONP(s)	Iron Oxide Nanoparticle(s)
IRF	Interferon Regulatory Factor
IRF3	Interferon Regulatory Factor 3
iRGD	Internalizing Arginine-Glycine-Aspartate Peptide
JAK	Janus Kinase
KRAS	Kirsten Rat Sarcoma Viral Oncogene Homolog
LLC	Lewis Lung Carcinoma
LNP(s)	Lipid Nanoparticle(s)
MDS	Myelodysplastic Syndrome
MDSC(s)	Myeloid-Derived Suppressor Cell(s)
MHC I	Major Histocompatibility Complex I
MHC II	Major histocompatibility complex class II
HPAA	Hyperbranched poly(amido amine)
MHT	Magnetic Hyperthermia
miRNA	MicroRNA
MM	Multiple Myeloma
MMP-2	Matrix Metalloproteinase-2
MMP-9	Matrix Metalloproteinase-9
MOF	Metal–Organic Framework
mPEG	Methoxy Polyethylene Glycol
MPS	Mononuclear Phagocyte System
mRNA	Messenger RNA
MRI	Magnetic Resonance Imaging
mTOR	Mammalian Target of Rapamycin
MyD88	Myeloid Differentiation Primary Response 88
N-BP	Nitrogen-Containing Bisphosphonate
NBCD(s)	Non-Biological Complex Drug(s)
ncRNA	Non-Coding RNA
NF-κB	Nuclear Factor Kappa B
NIR	Near-Infrared
NK	Natural Killer (Cell)
mPLA	monophosphoryl lipid A
NOD/SCID	Non-Obese Diabetic/Severe Combined Immunodeficient
NP(s)	Nanoparticle(s)
NSG	NOD Scid Gamma
NSCLC	Non-Small Cell Lung Cancer
OPN	Osteopontin
PBA	Phenylboronic Acid
PBMC(s)	Peripheral Blood Mononuclear Cell(s)
PD-1	Programmed Cell Death Protein 1
PD-L1	Programmed Death-Ligand 1
PDT	Photodynamic Therapy
PEG	Polyethylene Glycol
PEI	Polyethylenimine
PET	Positron Emission Tomography
PLGA	Poly(Lactic-co-Glycolic Acid)
PMOA	Primary Mode of Action
poly(I:C)	Polyinosinic:Polycytidylic Acid
PSMA	Prostate-Specific Membrane Antigen
PTHrP	Parathyroid Hormone-Related Protein
PTT	Photothermal Therapy
PTX	Paclitaxel
QbD	Quality by Design
RANKL	Receptor Activator of Nuclear Factor Kappa-B Ligand
RBC	Red Blood Cell
RES	Reticuloendothelial System
RGD	Arginine-Glycine-Aspartate
ROS	Reactive Oxygen Species
sgRNA	Single-Guide RNA
SHH	Sonic Hedgehog
Siglec-15	Sialic Acid-Binding Immunoglobulin-Like Lectin 15
siRNA	Small Interfering RNA
SIRS	Systemic Inflammatory Response Syndrome
SPION(s)	Superparamagnetic Iron Oxide Nanoparticle(s)
STAT1	Signal Transducer and Activator of Transcription 1
STAT4	Signal Transducer and Activator of Transcription 4
STING	Stimulator of Interferon Genes
TAM(s)	Tumor-Associated Macrophage(s)
TBK1	TANK-Binding Kinase 1
TGF-β	Transforming Growth Factor Beta
Th1	T Helper 1 (Cell)
TIM-3	T-cell Immunoglobulin and Mucin-Domain Containing-3
TLR	Toll-Like Receptor
TNF-α	Tumor Necrosis Factor Alpha
Treg(s)	Regulatory T-cell(s)
TRIF	TIR-Domain-Containing Adapter-Inducing Interferon-Beta
µCT	Micro-Computed Tomography
ZEB1	Zinc Finger E-Box Binding Homeobox 1
ZIF-8	Zeolitic Imidazolate Framework-8
ZOL	Zoledronate
